# Spatially Varying Drivers of Temporal β Diversity in Forest Avian Communities

**DOI:** 10.1002/ece3.72359

**Published:** 2025-10-22

**Authors:** Jess Dong, Stephen N. Matthews, Matthew B. Shumar, William E. Peterman

**Affiliations:** ^1^ School of Environment and Natural Resources Ohio State University Columbus Ohio USA

**Keywords:** β diversity, biodiversity, climate change, forests, LULC, spatial heterogeneity

## Abstract

Preserving biodiversity is crucial for ecosystem function, necessitating its measurement and monitoring to comprehend human impacts on the environment. β‐diversity, a key metric, can be used to measure changes in community diversity and assemblages over time. Multiple lines of evidence show that climate, land use land cover (LULC), and topography independently influence diversity; more recent work also shows that LULC can decouple climate effects, creating refugia. Spatial structures also affect the relationships between ecological processes and environmental factors. This study uses two discrete generations of breeding bird atlas data in Ohio, 24 years apart, to investigate the effects of environmental changes and spatial heterogeneity on statewide forest bird community diversity. Utilizing machine learning, we identified elevation variability, mean forest patch size, and total forest area as the most significant variables affecting temporal β diversity dissimilarity (referred to as temporal dissimilarity). The smallest changes in temporal dissimilarity occurred in landscapes with increased mean forest patch size, forest class area, and varied elevation. Furthermore, the results revealed a weak interaction between total forest area and elevation variability, with the largest temporal dissimilarity occurring in sites with the highest loss of total forest area and invariable elevation. We pinpointed regions in Ohio experiencing the greatest amount of composition changes in intensified agricultural and developed lands. A geographically weighted regression model indicated that the relationships between temporal dissimilarity and elevation variability, mean forest patch size, total forest area, and annual maximum temperature varied spatially within the state. Our study integrates ecological patterns with spatial patterns, showing that landscapes can interact with climate to mitigate regional climatic impacts and suggest effective conservation areas. Our findings also indicate that the relationships between ecological processes and the environment are not always spatially consistent.

## Graphical abstract

Understanding how environmental changes and spatial heterogeneity influence biodiversity is crucial for conservation. Using Ohio Breeding Bird Atlas data and machine learning, we identified elevation variability and forest availability as key drivers of β diversity dissimilarity, with the greatest assemblage changes in areas with high total forest loss and low elevation variability. Our findings highlight spatially non‐stationary relationships between ecological processes and environmental factors, emphasizing the importance of integrating landscape and climatic interactions for effective conservation planning.

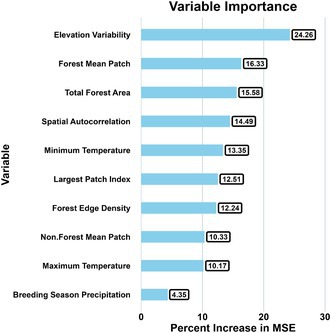

## 1 Introduction

Biodiversity is fundamental to ecosystem function, enhancing resource use efficiency and resistance to disturbance (Cardinale et al. 2012; Loreau and De Mazancourt 2013). Anthropogenic activities, such as global warming and habitat destruction, significantly impact biodiversity, necessitating its measurement and monitoring. Beta diversity (β), which is critical for understanding ecosystem functions, is especially useful for comparing species composition across multiple ecosystem communities and tracking temporal variations in species assemblages at specific sites (Whittaker 1960, 1972; Socolar et al. 2016). Temporal β diversity changes (referred to as temporal dissimilarity) reveal the changes in species assemblage over time, offering key insights into how the community might reorganize itself in response to environmental changes and natural disturbance (Lamy et al. 2015). Such information is essential for understanding the resistance and resilience of the system. However, predicting changes in temporal dissimilarity requires understanding the complex environmental drivers that shape species distribution.

Environmental changes drive species distribution in many ways (Whittaker 1972; Clarke and Gaston 2006; Cornell and Harrison 2014; Cadotte and Tucker 2017). Climate change alters species distribution, as warming and changes in precipitation challenge resource availability for flora and fauna. Land use and land cover (LULC) significantly influence species distribution by altering the composition and configuration of suitable habitat (Jiguet et al. 2005; Coops et al. 2009; Newbold et al. 2015; Regos et al. 2018). Habitat loss has led to fragmented landscapes, making LULC a limiting factor in species distribution (Jarzyna et al. 2015). For instance, Regos et al. (2018) showed that land‐use change is a strong predictor of bird species distribution in boreal forests (southern Quebec), linking land cover conversion and intensification to declining bird species. Globally, land use changes reduce species richness and total abundance (Newbold et al. 2015).

Critically, climate change and LULC interact to influence species occurrence and distribution, with landscape heterogeneity mediating or intensifying climatic impacts (Jarzyna et al. 2015; Newbold et al. 2019; Northrup et al. 2019). Climatic decoupling occurs when landscape heterogeneity creates local climatic variations and refugia that buffer regional climate change (Oliver et al. 2010; Dobrowski 2011; Hampe and Jump 2011; Frey et al. 2016; Lenoir et al. 2017; Suggitt et al. 2018). Forests can create areas that are shielded from regional climatic limitations by maintaining water balance and lowering exposure to sun (Lenoir et al. 2017; Greiser et al. 2020). The buffering capacity of forests is especially prominent in dense and old‐growth forests because of the tall canopy covers, high biomass, heterogeneous vertical structures (Frey et al. 2016; Betts et al. 2018; Greiser et al. 2020). Furthermore, Suggitt et al. (2018) found that microrefugia formed by topographic variation reduced the likelihood of plant and insect extirpation, but only when warming negatively affected species, showing climate–landscape interactions. Together, woody plants and heterogeneous topography can offer climatic protection, making forest and topographic complexity essential for understanding species' responses to climate change.

Furthermore, ecological processes are inherently spatial. Tobler's first law of geography posits that everything is related, but nearer things are more related than distant ones (Tobler 1970). Spatial relationships impact ecological processes by interacting with environmental factors. Spatial heterogeneity, in which relationships between ecological variables vary by spatial context, is key to understanding these dynamics. This non‐stationarity, a change in relationship between variables (Bueno de Mesquita et al. 2021), implies that the influence of environmental factors on ecological processes changes across spatial scales and locations (i.e., spatial variation of the species‐environment relationship). For instance, the distribution of plant species in a heterogeneous landscape is affected by spatially variable factors, such as soil type, moisture, and nutrient availability, resulting in distinct plant communities in different areas (Legendre and Fortin 1989).

Birds, a highly mobile species, are sensitive to environmental change (Bellard et al. 2012; Bateman et al. 2020) and have been intensively studied (Donaldson et al. 2017). Tracking spatiotemporal changes in their occurrence has the potential to inform species' resilience to climate and LULC changes, aiding management efforts. This is especially important for the avian communities in the Eastern Temperate Forest (ETF) of the U.S., a region that faces compounding climate and landscape changes. Climate shifts (warmer, wetter, more variable conditions) are altering phenology and vegetation competition, cascading to wildlife survival and invasive species spread (Gilliam 2016; Rustad et al. 2012). Meanwhile, land‐use change—the second‐largest driver of ecosystem change after climate—has reduced and fragmented ETF since European colonization through cropland conversion, urbanization, and logging (Fischer et al. 2013; Riitters et al. 2012; Sala et al. 2000). Despite improved knowledge, we lack a clear understanding of how the interacting effects of climate and LULC change drive patterns of change in avian communities. Moreover, research has primarily focused on biodiversity trends at large scales, whereas management is implemented locally, and it is uncertain whether local community dynamics align with global trends (Díaz and Malhi 2022). Understanding these interactions is essential for predicting species and ecosystem responses to environmental changes, and for developing effective conservation strategies (Levin 1992; Wiens 2002; Turner 2005; Badgley et al. 2017). To address these knowledge gaps, we utilize Ohio Breeding Bird Atlas data to (1) investigate whether and how environmental changes and topography influence the temporal changes in β diversity (i.e., temporal dissimilarity) in forest avian communities, (2) explore interacting effects of climate and LULC on temporal dissimilarity, and (3) examine the spatial heterogeneity of the diversity‐environment relations at the state level.

## 2 Methods and Materials

### 2.1 Study Design and Bird Data Collection

**FIGURE 1:**
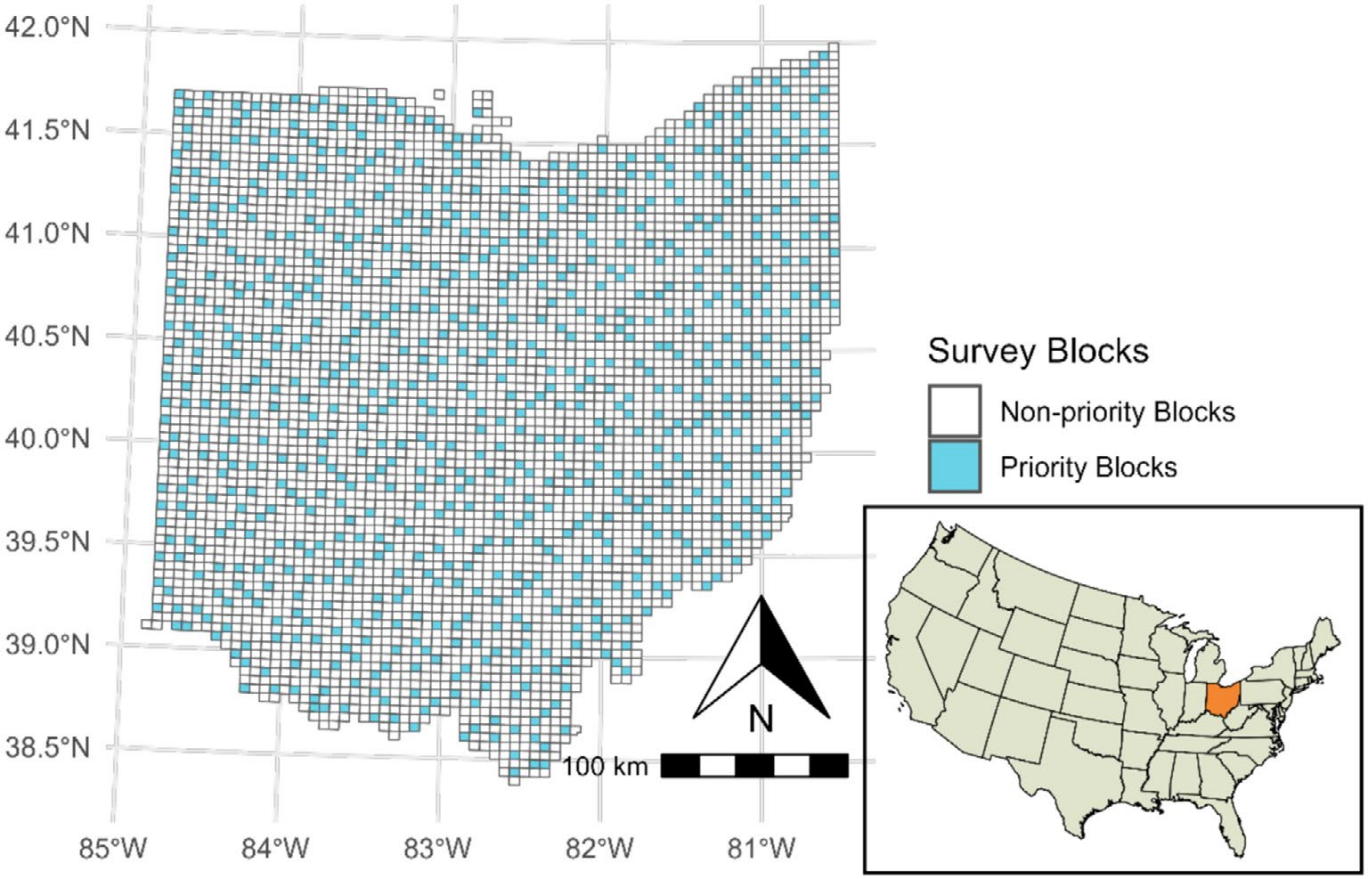
Ohio Breeding Bird Atlas (OBBA) sample design. The state of Ohio was divided into a total of 4437 (24.5 km^*2*^) blocks. The priority blocks (in blue color) were surveyed during the first atlas (OBBA 1, 1982–1986) and all blocks were surveyed during the second atlas (OBBA 2, 2006–2011).

The Ohio Breeding Bird Atlas (OBBA) was a statewide effort to inventory the distribution and population of bird species across Ohio (Rodewald et al. 2016). There have been two generations of breeding bird atlases in Ohio, with the first atlas (OBBA1) occurring from 1982 to 1987, and a second atlas (OBBA2) from 2006 to 2011. Each year, occurrence data were collected during the breeding season. The entire state was divided into 4.9 km × 4.9 km blocks (24.5 km^2^, UTM datum), following the standard U.S. Geological Survey 7.5‐min topographic mapping scheme (see Rodewald et al. 2016, figure 3.2.1). Blocks located along the border of the state were excluded if only a small portion of the area was within Ohio. For OBBA1, a total of 764 blocks (priority blocks, Figure 1) were selected with a stratified random sampling design, wherein one block within each topographic map was randomly selected to be surveyed for occurrence. Coverage was expanded during OBBA2, and all six blocks per topographic map (*n* = 4437) were surveyed. For this study, however, we only used data from the priority blocks, as they were consistently surveyed during both periods.

Each of the atlas generations employed a set of carefully designed breeding evidence codes that were grouped into breeding confirmation levels (i.e., observed, possible, probable, and confirmed). Slight modifications were applied to the codes used for OBBA2 in order to standardize them and provide consistency with other state atlas projects. Sixteen OBBA2 breeding evidence codes aligned with those used during OBBA1 and allowed us to assess block‐level changes. To facilitate comparison between the atlas generations, coordinators set targets for the number of effort hours and species totals for each priority block. The median survey effort was 24 and 20 h per priority block for OBBA1 and OBBA2, respectively. The average number of species recorded per effort hour was similar during each period. To differentiate transient individuals from locally breeding birds, safe dates were developed and used in combination with breeding evidence codes for each species (see Rodewald et al. 2016 for details).

Excluding the most ubiquitous species, the presence/absence of 82 forest bird species was recorded during both sampling periods, with forest birds broadly defined as any species that fully or partially utilize woody‐plant habitats, including forest (mature, closed canopy), woodland (medium stature trees), and shrubland (full list in Table 1); habitat information was based on the widely used trait database AVONET (Tobias et al. 2022).

**TABLE 1:** List of the avian forest species surveyed in the Ohio Breeding Bird Atlas I & II (1982–1987 & 2006–2011).

Common name	Scientific name	Habitat
Acadian Flycatcher	*Empidonax virescens*	Forest
Alder Flycatcher	*Empidonax alnorum*	Shrubland
American Redstart	*Setophaga ruticilla*	Forest
American Woodcock	*Scolopax minor*	Shrubland
Baltimore Oriole	*Icterus galbula*	Forest
Barred Owl	*Strix varia*	Forest
Black‐and‐white Warbler	*Mniotilta varia*	Forest
Black‐billed Cuckoo	*Coccyzus erythropthalmus*	Forest
Blackburnian Warbler	*Setophaga fusca*	Forest
Black‐throated Green Warbler	*Setophaga virens*	Forest
Blue Grosbeak	*Passerina caerulea*	Shrubland
Blue‐gray Gnatcatcher	*Polioptila caerulea*	Woodland
Blue‐headed Vireo	*Vireo solitarius*	Forest
Blue‐winged Warbler	*Vermivora cyanoptera*	Shrubland
Broad‐winged Hawk	*Buteo platypterus*	Forest
Brown Creeper	*Certhia americana*	Forest
Brown Thrasher	*Toxostoma rufum*	Shrubland
Brown‐headed Cowbird	*Molothrus ater*	Shrubland
Carolina Wren	*Thryothorus ludovicianus*	Woodland
Cedar Waxwing	*Bombycilla cedrorum*	Woodland
Cerulean Warbler	*Setophaga cerulea*	Forest
Chestnut‐sided Warbler	*Setophaga pensylvanica*	Shrubland
Cooper's Hawk	*Accipiter cooperii*	Woodland
Dark‐eyed Junco	*Junco hyemalis*	Forest
Downy Woodpecker	*Picoides pubescens*	Woodland
Eastern Bluebird	*Sialia sialis*	Woodland
Eastern Kingbird	*Tyrannus tyrannus*	Woodland
Eastern Phoebe	*Sayornis phoebe*	Woodland
Eastern Screech‐Owl	*Megascops asio*	Woodland
Eastern Towhee	*Pipilo erythrophthalmus*	Shrubland
Eastern Whip‐poor‐will	*Antrostomus vociferus*	Woodland
Eastern Wood‐Pewee	*Contopus virens*	Forest
Great Crested Flycatcher	*Myiarchus crinitus*	Woodland
Great Horned Owl	*Bubo virginianus*	Forest
Hairy Woodpecker	*Leuconotopicus villosus*	Woodland
Hermit Thrush	*Catharus guttatus*	Forest
Hooded Warbler	*Setophaga citrina*	Woodland
House Finch	*Haemorhous mexicanus*	Shrubland
House Wren	*Troglodytes aedon*	Shrubland
Kentucky Warbler	*Geothlypis formosa*	Woodland
Lark Sparrow	*Chondestes grammacus*	Shrubland
Least Flycatcher	*Empidonax minimus*	Woodland
Louisiana Waterthrush	*Parkesia motacilla*	Forest
Magnolia Warbler	*Setophaga magnolia*	Forest
Northern Flicker	*Colaptes auratus*	Woodland
Northern Mockingbird	*Mimus polyglottos*	Shrubland
Northern Parula	*Setophaga americana*	Forest
Northern Waterthrush	*Parkesia noveboracensis*	Shrubland
Orchard Oriole	*Icterus spurius*	Woodland
Ovenbird	*Seiurus aurocapilla*	Forest
Pileated Woodpecker	*Dryocopus pileatus*	Forest
Pine Siskin	*Spinus pinus*	Forest
Pine Warbler	*Setophaga pinus*	Forest
Prairie Warbler	*Setophaga discolor*	Shrubland
Prothonotary Warbler	*Protonotaria citrea*	Woodland
Purple Finch	*Haemorhous purpureus*	Forest
Red‐bellied Woodpecker	*Melanerpes carolinus*	Woodland
Red‐breasted Nuthatch	*Sitta canadensis*	Forest
Red‐eyed Vireo	*Vireo olivaceus*	Forest
Red‐headed Woodpecker	*Melanerpes erythrocephalus*	Woodland
Red‐shouldered Hawk	*Buteo lineatus*	Woodland
Red‐tailed Hawk	*Buteo jamaicensis*	Woodland
Rose‐breasted Grosbeak	*Pheucticus ludovicianus*	Forest
Ruby‐throated Hummingbird	*Archilochus colubris*	Woodland
Scarlet Tanager	*Piranga olivacea*	Forest
Sharp‐shinned Hawk	*Accipiter striatus*	Forest
Summer Tanager	*Piranga rubra*	Woodland
Tufted Titmouse	*Baeolophus bicolor*	Forest
Veery	*Catharus fuscescens*	Forest
Warbling Vireo	*Vireo gilvus*	Shrubland
White‐breasted Nuthatch	*Sitta carolinensis*	Forest
White‐eyed Vireo	*Vireo griseus*	Shrubland
Willow Flycatcher	*Empidonax traillii*	Shrubland
Winter Wren	*Troglodytes hiemalis*	Forest
Wood Thrush	*Hylocichla mustelina*	Forest
Worm‐eating Warbler	*Helmitheros vermivorum*	Forest
Yellow Warbler	*Setophaga petechia*	Shrubland
Yellow‐bellied Sapsucker	*Sphyrapicus varius*	Woodland
Yellow‐billed Cuckoo	*Coccyzus americanus*	Forest
Yellow‐breasted Chat	*Icteria virens*	Shrubland
Yellow‐throated Vireo	*Vireo flavifrons*	Woodland
Yellow‐throated Warbler	*Setophaga dominica*	Forest

*Note:* Species that utilize forest, shrubland, and woodland as habitats are all considered to be part of the forest community in this study. Habitat information is extracted from AVONET. Forest = tall‐tree dominated vegetation with more or less closed canopy; woodland = medium stature tree‐dominated habitats; and shrubland = low stature bushy habitats.

### 2.2 Environmental and Spatial Variables

Climate variable information was generated by the Parameter‐elevation Regressions on Independent Slopes Model (PRISM) (Oregon Climate Service, Corvallis, Oregon, USA) at a 1‐km resolution. Given that seasonal temperatures and precipitation affect bird survival rates, breeding success, and distributions (reviewed by Leech and Crick 2007), these climate variables were included as annual temperature, annual maximum temperature, annual minimum temperature, spring temperature, annual precipitation, and breeding season precipitation. Annual maximum and minimum temperatures could be interpreted as summer and winter temperatures, respectively. We broadly defined May–August as the breeding season given the wide range of breeding dates of the species included. We took the 5‐year average values of these variables matching each bird sampling period. We checked variable collinearity using the *ggcorr* function of the “GGally” package (Schloerke et al. 2024, version 2.2.1) with 0.6 as the threshold, and excluded annual temperature, annual precipitation, and spring temperature (Figure S1). We calculated the change in the climate variable values between OBBA 1 and OBBA 2 as ∆variable = variable _OBBA2_—variable _OBBA1_. For example, ∆spring temperature = spring temperature _OBBA2_—spring temperature _OBBA1_.

Forest cover data from 1986 to 2010 were downloaded from the NACP North American Forest Dynamics Project: Forest Disturbance History from Landsat, 1986–2010 (Goward et al. 2016). This dataset (30 m resolution) has four classes: water, no forest, forest, and disturbance (disturbance over forest occurred that year). We used the *Setnull* function to exclude the disturbance class in ArcGIS Pro before extracting landscape metric values (ArcGIS Pro 3.0.0), as forest disturbance was beyond the scope of this study. There are two dimensions of landscape metrics: composition and configuration. Composition refers to the cover classes present, and configuration refers to the arrangements of these types. There are many ways to measure composition and configuration, among which connectivity has been shown to be an integral part of species range shift prediction (Dahirel et al. 2021), making fragmentation‐related metrics highly relevant to exploring the connection between landscape and bird occurrence. Several studies illustrated that mean patch size, edge density (ED), and largest patch index were relevant to bird diversity and density (Saveraid et al. 2001; Borges et al. 2017; Morelli et al. 2018). Hence, we selected two landscape‐level metrics: total edge (TE) and largest patch index (LPI), and three class‐level metrics: mean patch size (AREA_MN), total class area (CA), and ED. We extracted values using the *sample_lsm* function in the “landscapemetrics” package (Hesselbarth et al. 2019, version 1.5.5) in R (R Core Team 2023, version 4.3.2) for the landscape (in this case, the block) and each class within the landscape. Thus, for each block, there were two landscape measurements (i.e., total edge and LPI), and for each class (water, forest, non‐forest) within the block, there were three measurements (e.g., forest mean patch, total forest area, and forest edge density) (see Table S1 for details). Finally, we calculated the change in the metrics between OBBA1 and OBBA2 using the formula ∆metric = metric _OBBA2_—metric _OBBA1_. We selected two time points to evaluate landscape change, rather than continuous data across the entire period. Our ultimate goal was to understand how long‐term environmental changes might impact changes in diversity. So, our focus was to assess long‐term landscape change over a decadal scale, rather than year‐to‐year fluctuations. In this context, data from two time points were adequate to represent the broad‐scale landscape transitions and temporally aligned with the diversity dissimilarity.

The changes in the water class between 1986 and 2010 were small for all metrics (∆Water < 0.01); thus, we excluded that class entirely. We checked variable collinearity using the *ggcorr* function of the “GGally” package (Schloerke et al. 2024, version 2.2.1) with 0.6 as the threshold, and excluded total edge, non‐forest edge density, and total non‐forest area, as they were highly correlated with multiple other variables (Figure S1). Therefore, the final set of environmental variables included the change in annual maximum temperature, annual minimum temperature, breeding season precipitation, LPI, forest mean patch size, non‐forest mean patch size, total forest area, and forest edge density (see Table S2 for details).

To account for spatial autocorrelation, we used Moran's Eigenvector Maps (MEM) approach to generate the spatial variable (Dray et al. 2006). First, we extracted the centroids of each site using the package “sf” (Pebesma 2018, version 1.0.15). Because all sites were 4.9 × 4.9 km square blocks, shape did not play a role. Then, we defined neighbors based on Gabriel graphs and created a weight matrix using the inverse of distance between neighbors with the “spdep” package (Bivand and Wong 2018, version 1.3.1). Lastly, we computed MEMs as the spatial variable using the “adespatial” package (Dray et al. 2023, version 0.3.23). All computations were done in R (R Core Team 2023).

To standardize elevation for comparison, we computed the coefficient of variation (CV) in each site. The digital elevation data were downloaded from Earth Explorer (https://earthexplorer.usgs.gov/) by selecting the boundary of Ohio, USA. We used the STRM 1 Arc‐Second Global dataset and used all tiles that covered the state. Two tiles in Meigs County (southeast corner) were missing, which were supplemented by data from the Ohio Geographically Referenced Information Program (https://das.ohio.gov/technology‐and‐strategy/OGRIP/data_services). All tiles were stitched together into one raster in ArcGIS Pro using the *Mosaic to New Raster* function (ArcGIS Pro 3.0.0). All rasters used the WGS1984 geographic coordinate system at the 0.000278‐degree resolution. In R (R Core Team 2023, version 4.3.2), first, we calculated the mean and standard deviation of elevation in each site using the “terra” package (Hijmans 2023, version 1.7.65). Finally, we calculated the CV of elevation, termed as elevation variability. Unlike the environmental covariates, we did not calculate change statistics for spatial autocorrelation and elevation variability between sample periods because these measures are largely static (see Table S2 for range values).

### 2.3 Statistical Analysis

#### 2.3.1 Community Analysis (Objective 1)

At the community level, we first performed nonmetric multidimensional scaling (NMDS) to visualize the temporal shifts of Ohio bird communities in a two‐dimensional space, selecting the Bray–Curtis dissimilarity index and using the “vegan” package (stress = 0.187, Oksanen et al. 2022, version 2.6.4, Figure S2). ANOVA test of similarities (ANOSIM), with the *anosim* function of the “vegan” package (Oksanen et al. 2022), indicated that bird species diversity experienced a slight change between the two sampling periods (*p* = 0.001, *R* = 0.18); thus, we proceeded to quantify this temporal change. We used the *beta.temp* function of the “betapart” package (Baselga et al. 2023, version 1.6) to quantitatively measure the differences of β diversity of the same site in two different periods, using the occurrence data from the two sampling periods. The resulting value indicated the temporal diversity dissimilarity of the same site, which we call temporal dissimilarity. We selected the Jaccard dissimilarity index to place equal weights on shared and unique species. Jaccard dissimilarity ranges from 0 to 1 (unitless), with zero indicating no temporal changes in species composition and one indicating a complete change of composition.

#### 2.3.2 Spatial Dependence of Temporal Dissimilarity

Because spatial dependence can confound the interpretation of ecological processes, it was necessary to test whether spatial dependence played a role in the patterns of temporal dissimilarity. To investigate the spatial dependence of temporal dissimilarity, we first quantified the existence of spatial dependence with the global Moran's I index. We defined the neighborhood with Gabriel graphs and used the inverse distance weight matrix. We then used the *moran.test* function of the “spdep” package (Bivand and Wong 2018, version 1.3.1) to compute the Moran's I value. Global Moran's I ranges from −1 to 1. Negative one indicates perfect spatial dispersion, meaning that neighboring blocks have dissimilar values; whereas positive one indicates perfect spatial clustering, meaning that neighboring blocks have similar values. Zero indicates random spatial patterns. Once the global Moran's I index indicated spatial clustering (*I* = 0.26, *p* < 2.2e‐16), we further investigated the patterns of spatial clustering with the local Moran's I index (local indicator of spatial autocorrelation, LISA). This step was to identify and locate statistically significant clusters of blocks sharing similar values. We used the *localmoran* function of the “spdep” package (Bivand and Wong 2018, version 1.3.1). The global Moran's I evaluates statewide spatial dependence, while LISA detects localized clusters of high or low values driving these patterns. Together, they reveal that spatial connectivity, independent of ecological processes, influences the observed trends.

#### 2.3.3 Dissimilarity‐Environment Relationship Modeling (Objective 1 &2)

We used Random Forest (RF, Breiman 2001) to model the relationships between temporal dissimilarity and potential environmental stressors (i.e., changes in environmental metrics). Random Forest is a widely used machine learning method, which considers high‐order interactions between environmental variables and can automatically adjust to nonlinear relationships (Evans et al. 2011). This is an appropriate method for exploring the basic community structures with limited presumption about the relationships between species occurrence and the environment. We used the entire dataset without specifying a training set, because as an ensemble learning algorithm, RF bootstraps a subset (~63%) of the data randomly to build the trees. To find the optimum number of variables selected at each split, we first ran the model with default settings, then we used the *tuneRF* function from the “randomForest” package (Liaw and Wiener 2002, version 4.7.1.1) to determine the number of variables that minimized out‐of‐bag error (OOB). Finally, we decided on a model with two variables selected at each split and 500 trees, followed by model performance metrics (i.e., OOB) and univariable importance measurements (i.e., % Increase in MSE). In RF, variable importance is measured by the percentage increase in mean standard error (MSE) when a variable is being permuted; the greater the percentage increase in MSE, the greater the influence of the variable. We modeled the temporal dissimilarity as a function of changes in annual maximum temperature, annual minimum temperature, breeding season precipitation, LPI, forest mean patch size, non‐forest mean patch size, total forest area, and forest edge density, elevation variability, and spatial autocorrelation.

One of the limitations of RF is variable interaction measurements. Although RF can compute how the split of a given variable affects the split of another one, this is limited to pairs of variables. Thus, for exploratory studies with limited assumptions, the computational power required to test variable interactions is substantial. Here, we used the “diversityForest” package (Hornung and Wright 2023, version 0.4.0) to measure potential variable interactions of our dataset. Specifically, the *interactionfor* function was used to build models for interactions. The output of the model included the ranked variable pairs in ascending order based on *p* values of the interaction term. To visualize the variable interactions, we computed the partial dependence plot with the *partial* function of the “pdp” package (Greenwell 2017, version 0.8.1), then interpolated the model values using the *interp* function of the “akima” package (Akima and Gebhardt 2022, version 0.6–3.4); finally, we created the 3‐D graphs with the *plot_ly* function from the “plotly” package (Sievert 2020, version 4.10.4).

#### 2.3.4 Spatial Heterogeneity Modeling (Objective 3)

Spatial heterogeneity describes a non‐stationarity in the species‐environment relationship, where the relationship between variables varies based on location. This non‐stationary relationship implies that the influence of environmental factors on ecological processes changes across spatial localities. To test the spatial heterogeneity of temporal dissimilarity, we first fit an ordinary least squares model (OLS) with temporal dissimilarity as a function of the variables in the RF model above, excluding spatial autocorrelation. Then, we checked the OLS residuals for signs of spatial heterogeneity. OLS assumes the variance of the residual to be constant (homoskedasticity), meaning that the error structure is constant across different spatial locations. Unequal variance of the residual (heteroskedasticity), thus, can be a sign of spatial heterogeneity as the error structure is inconsistent across different spatial locations. Therefore, we quantitatively tested and confirmed the residual heteroskedasticity with the Breusch‐Pagan Test (*p* = 0.03), suggesting the possibility of spatial heterogeneity. We used a geographically weighted regression (GWR) model to understand the spatially varying relationships between temporal dissimilarity and the variables under consideration, as GWR allows parameters to vary locally. A Shapiro test indicated that the OLS residuals were not normally distributed (*p* < 0.01), right skewed by outliers. Therefore, we used the robust GWR model, which was designed to be less sensitive to outliers than the traditional GWR. We chose an adaptive weighting scheme because, due to the stratified randomization process, some blocks were closer to each other than others, especially those on the state boundary. The adaptive approach allowed for flexibility and adjustment of weights. We used the bi‐square kernel density function. The weighting scheme and kernel density would influence how the dataset was split into various subsets that represented different spatial locations. The model was fitted using the *gwr.robust* function of the “GWmodel” package, after which we adjusted the t values using the *gwr.t.adjust* function of the same package (Lu et al. 2014; Gollini et al. 2015, version 2.3.2). We used the adjusted t values because GWR models could increase the risk of Type I error by fitting into several subsets of the data. Adjusting t values typically is done by setting a higher critical value to account for the inflated risk. The robust GWR model included the F‐test for the parameter significance (i.e., Leung et al. test, Leung et al. 2000). The structure of this spatially explicit GWR model was the same as that of the OLS model: temporal dissimilarity as the function of the changes in annual maximum temperature, annual minimum temperature, breeding season precipitation, LPI, forest mean patch size, non‐forest mean patch size, total forest area, forest edge density, and elevation variability.

## 3 Results

**FIGURE 2:**
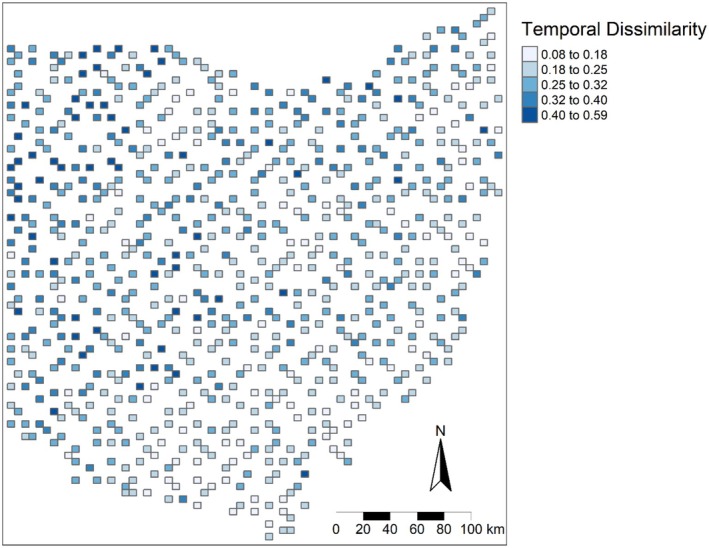
Map of β diversity temporal dissimilarity for 764 survey blocks in Ohio. Dissimilarity was calculated with the Jaccard coefficient by computing the temporal changes in β diversity for each block across 24 years (between 1982 and 1987 and 2006 and 2011). Dissimilarity ranges between zero and one (unitless). Low values indicate none to minimal changes in species assemblage, and high values suggest species composition shifts.

The community analysis showed that temporal dissimilarity ranged between 0.08 and 0.59 (mean = 0.27, Figure 2, Figure S3). The highest dissimilarity (0.3–0.59) values occurred near northern, southwestern, and northwestern Ohio (Figure 2).

**FIGURE 3:**
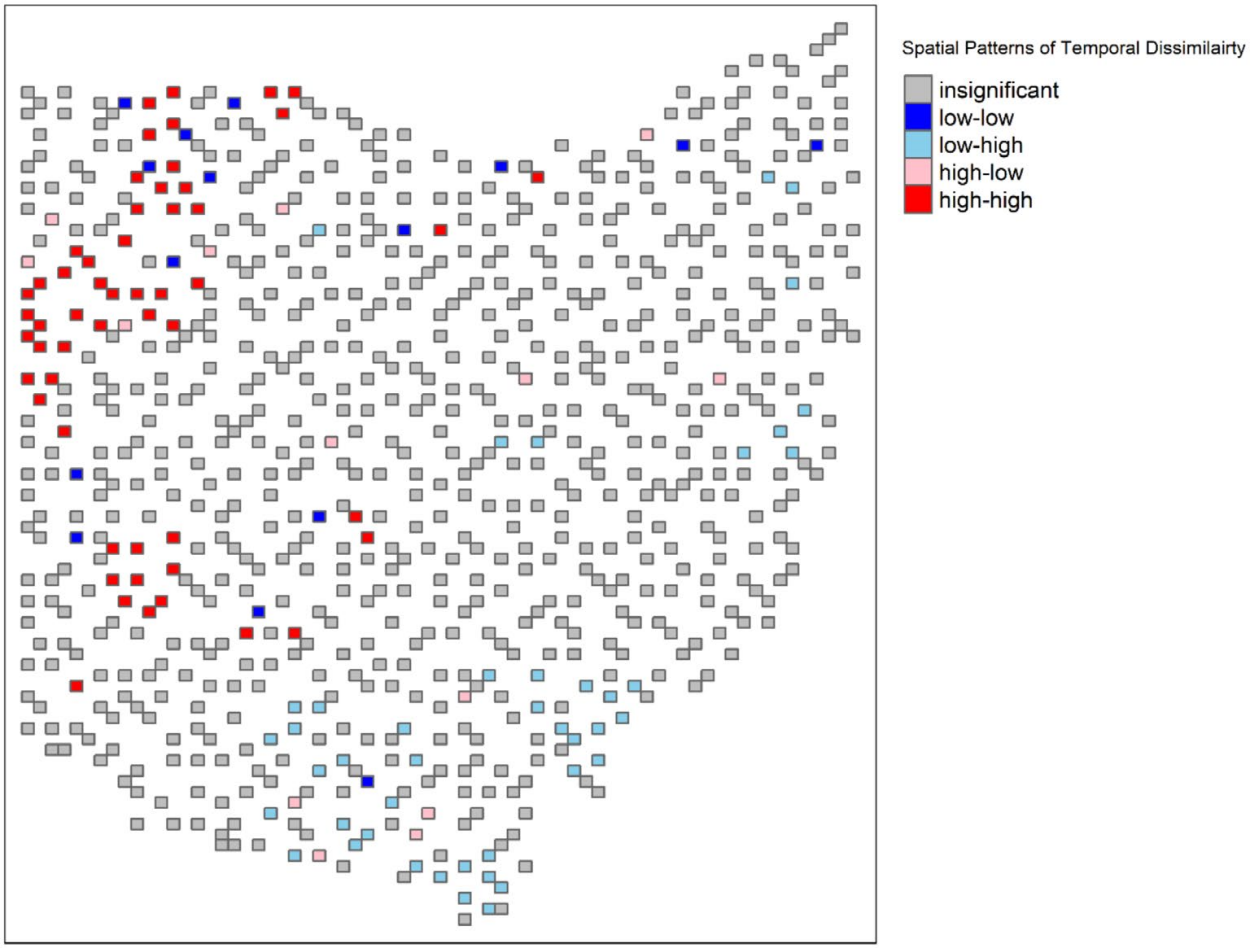
Locate Indicator of spatial autocorrelation (LISA) of β diversity temporal dissimilarity in Ohio. The high‐high (red) and low‐low (dark blue) cells indicate that the focal block and their neighboring blocks share similarly high and low dissimilarity values, respectively. The high‐low (pink) and low‐high (light blue) cells suggest that the focal block and their neighboring blocks have different levels of dissimilarity, either the focal block has high and their neighbors have low dissimilarity or vice versa.

**FIGURE 4:**
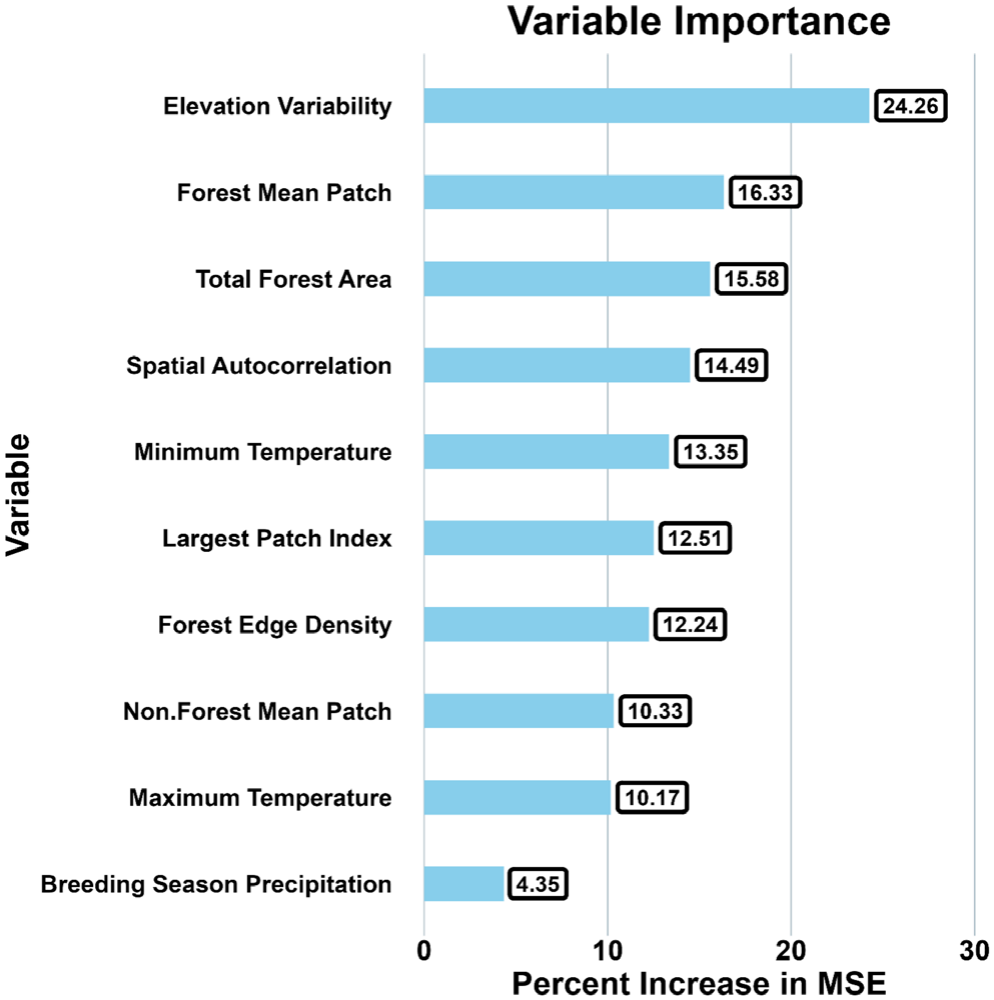
Variable importance of a Random Forest model. Percent Increase in MSE = increase of Mean Standard Error when a variable is being permuted. The higher the Percent Increase in MSE, the more important the variable is. Climate and landscape variables were calculated as the difference between OBBA1 and OBBA2 (V_i_ = V_OBBA2_—V_OBBA1_). Elevation variability was computed as the coefficient of variation of elevation in each block. Spatial autocorrelation was the Moran's eigenvector value for each block. The dependent variable was temporal dissimilarity of β diversity, which was computed based on data from Ohio Breeding Bird Atlas in 1982–1986 and 2006–2011.

**FIGURE 5:**
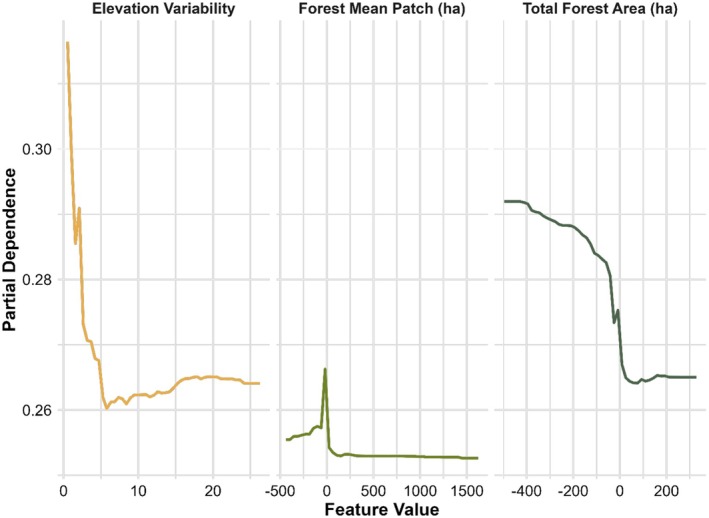
Partial dependence plots of the top three important variables of a Random Forest model for forest avian community in Ohio. *X*‐axis indicates the elevation variability (left) and changes in forest mean patch size (middle) and total forest area (right) between 1986 and 2010. Y‐axis shows the temporal dissimilarity of β diversity in 764 blocks between 1982–1987 and 2006–2011.

Once the global Moran's I suggested spatial clustering patterns of the temporal dissimilarity (*I* = 0.26), the local Moran's I index (LISA) confirmed that the statistically significant clusters of high temporal dissimilarity blocks were located in the western part (northwestern and southwestern) of Ohio (Figure 3, red blocks). There were several focal blocks with low temporal dissimilarity values spread across the state, but they did not form distinct clusters, as their neighboring blocks had insignificant values (Figure 3, dark blue blocks). Thus, the main contributors of the global spatial clustering patterns were the groups of high temporal dissimilarity blocks in western Ohio. The Random Forest model revealed that elevation variability was the most influential factor shaping forest bird community composition, followed by changes in mean forest patch size and total forest area (OOB = 0.005, pseudo *R* ^2^ = 24.09%, Figure 4). Partial dependence analyses showed consistent relationships between temporal dissimilarity and landscape variables (Figure 5). Temporal dissimilarity was highest in areas with homogeneous elevation but decreased as elevation variability increased. Larger mean forest patch sizes were associated with lower temporal dissimilarity. Finally, temporal dissimilarity displayed an inverse relationship with changes in total forest area, reaching its highest levels when forest area was most reduced.

A weak interaction between elevation variability and changes in total forest area was detected (*p* = 0.06, Figure S4). Temporal dissimilarity was the highest when most total forest areas were lost in the least varied elevation. For a given elevation variability, temporal dissimilarity was lower when total forest areas were gained (Figure S4).

**FIGURE 6:**
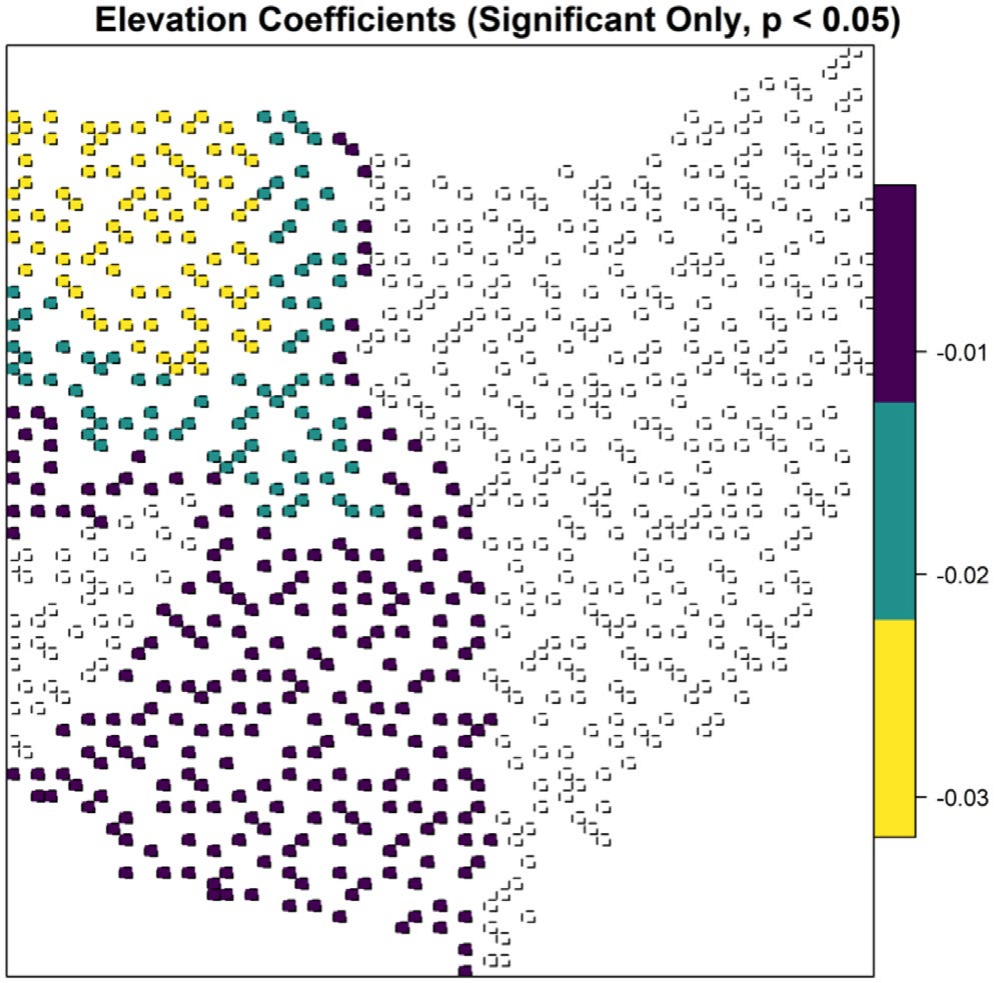
Robust geographically weighted regression model for the relationship between temporal dissimilarity of β diversity and elevation variability, for the forest avian community in Ohio. Locations with statistically significant spatial heterogeneity are shown and colors represent varying parameter coefficient values, ranging from low magnitude (purple) to high magnitude (yellow).

**FIGURE 7:**
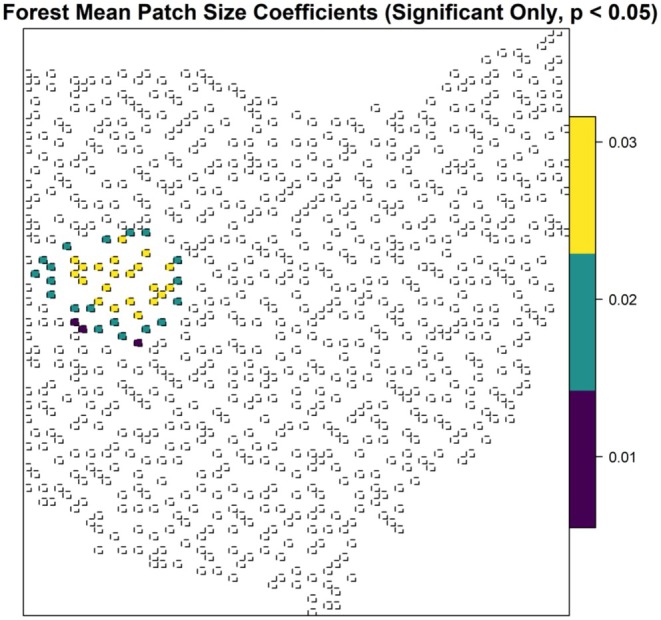
Robust geographically weighted regression model for the relationship between temporal dissimilarity of β diversity and forest mean patch, for the forest avian community in Ohio. Locations with statistically significant spatial heterogeneity are shown and colors represent varying parameter coefficient values, ranging from low magnitude (purple) to high magnitude (yellow).

**FIGURE 8:**
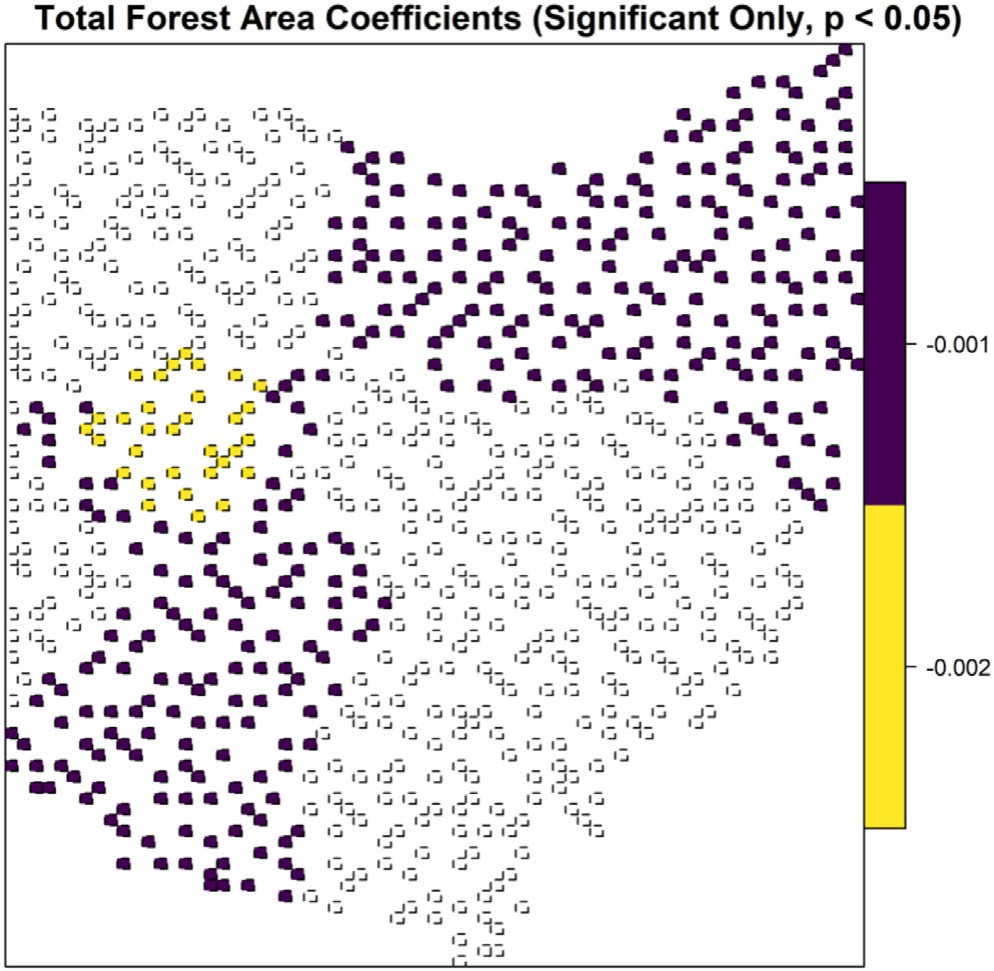
Robust geographically weighted regression model for the relationship between temporal dissimilarity of β diversity and total forest area, for the forest avian community in Ohio. Locations with statistically significant spatial heterogeneity are shown and colors represent varying parameter coefficient values, ranging from low magnitude (purple) to high magnitude (yellow).

**FIGURE 9:**
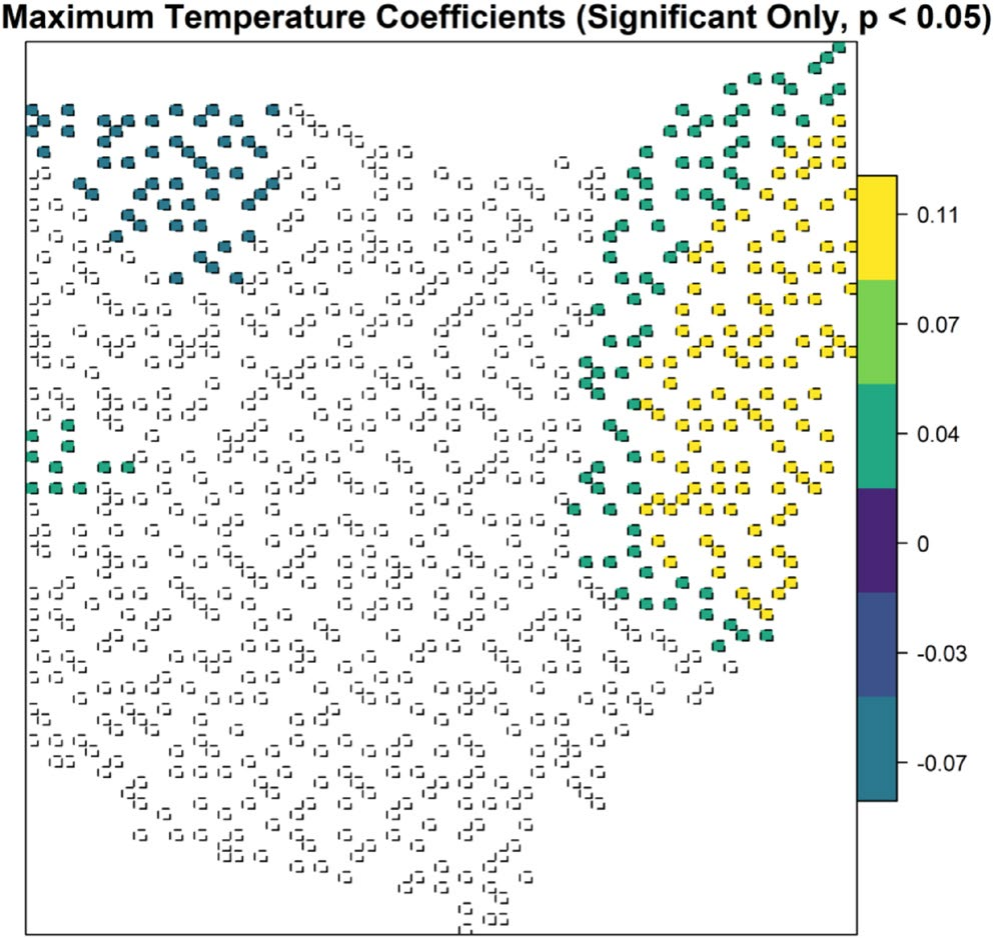
Robust geographically weighted regression model for the relationship between temporal dissimilarity of β diversity and annual maximum temperature, for the forest avian community in Ohio. Locations with statistically significant spatial heterogeneity are shown and colors represent varying parameter coefficient values, ranging from negative (blue‐purple) to positive (green‐yellow).

The robust GWR model demonstrated spatial heterogeneity in the relationships between temporal dissimilarity and elevation variability (*p* < 0.001), changes in forest mean patch (*p* = 0.003), total forest area (*p* = 0.02), and annual maximum temperature (*p* < 0.001, Table 2). There was an inverse relationship between temporal dissimilarity and elevation variability with varying magnitudes, but it was only statistically significant in western Ohio (Figure 6). Changes in forest mean patch size had a positive correlation with temporal dissimilarity with varying magnitudes, but this relationship was only statistically significant in a cluster of blocks in western Ohio (Figure 7). Changes in total forest area were inversely correlated with temporal dissimilarity in southwestern and northeastern Ohio, with a slightly steeper slope in a cluster of blocks in western Ohio (Figure 8). In northwestern Ohio, changes in annual maximum temperature had a negative correlation with temporal dissimilarity; whereas in eastern Ohio, along with a cluster of 10 blocks in western Ohio, this relationship was positive (Figure 9).

**TABLE 2:** Parameter estimates of the OLS model as well as parameter estimates and significant tests of the GWR model.

Variable	OLS coefficient	Robust GWR Coefficient					Leung et al. test (*p*)
		Min	Q1	Median	Q3	Max	
Maximum Temperature	0.023	−0.068	0.009	0.043	0.063	0.107	**< 0.001** ***
Minimum Temperature	0.028	−0.035	0.006	0.019	0.028	0.050	1.00
Breeding Season Precipitation	0.0001	−0.0004	0.000006	0.0002	0.0003	0.0004	0.29
Non‐Forest Mean Patch	−0.00001	−0.0004	−0.00002	−0.000007	0.00001	0.0001	0.26
Total Forest Area	−0.0003	−0.0021	−0.0004	−0.0003	−0.0001	0.0002	**0.02** *
Forest Edge Density	−0.003	−0.0098	−0.004	−0.002	−0.001	0.006	0.20
Forest Mean Patch	−0.00002	−0.0007	−0.00003	0.00003	0.0011	0.030	**0.003** **
Largest Patch Index	−0.000003	−0.0026	−0.0005	0.00006	0.0009	0.004	0.64
Elevation Variability	−0.005	−0.025	−0.008	−0.004	−0.001	0.001	**< 0.001** ***
Intercept	0.286	0.224	0.244	0.271	0.309	0.393	< 0.001***
Adj *R* ^2^	1.16			0.23			

*Note:* The robust GWR model was used to investigate the spatial heterogeneity of the temporal dissimilarity–environment relationships of the forest avian community in Ohio. Temporal dissimilarity was computed with the Jaccard index, based on the occurrence data from the Ohio Breeding Bird Atlas in 1982–1987 and 2006–2011. The spatial variability of elevation variability, forest mean patch, total forest area, and maximum temperature was statistically significant in Leung et al. test (*F* test, *p* values in bold).

*p < 0.05, **p < 0.01, ***p < 0.001.

## 4 Discussion

Many argued that in the age of the Anthropocene, biodiversity studies should move beyond simply measuring richness toward tracking compositional changes (Dornelas et al. 2014; Hillebrand et al. 2018; Magurran et al. 2019; Chao et al. 2023). Measuring temporal β diversity provides critical insights into these dynamics. Shifts in assemblages highlight how communities respond to changes in ecological processes, whether deterministic or stochastic. Therefore, temporal dissimilarity serves as an indirect measure of community stability and resilience. Our first objective was to test whether the Ohio forest avian communities experienced compositional change and, if so, what roles environmental changes and topography played. We found that the forest bird assemblage indeed experienced moderate change between the 1980s and 2000s (mean temporal dissimilarity = 0.27), consistent with patterns observed in other regions and taxa (Dornelas et al. 2014; Tsianou et al. 2021; Wu et al. 2022). Given that temporal dissimilarity ranges from 0 to 1, blocks with values between 0.3 and 0.59 exhibited high composition change, indicating that 30%–59% of the community assembly shifted over time, with the greatest shifts occurring in heavily modified agricultural and urban landscapes. The changes in forest species composition were inversely linked to elevation variability, changes in mean forest patch size, and changes in total forest area, all of which were shaped by geographical histories that affected processes that generated biodiversity (Tolmos et al. 2024). These results also supported our second objective to understand that landscape, particularly change in forest cover and topographic complexity, could decouple the climatic effects serving as potential ecological refugia. Our third objective was to detect spatial heterogeneity of diverse‐environment relationships, and we found support for varied spatial relationships between forest bird community composition shifts and environmental factors. The inverse relationship between composition change and elevation variation was prominent in western Ohio. Changes in total forest area were inversely associated with species composition changes in western and northeastern Ohio. In northwestern Ohio, rising annual maximum temperatures correlated with fewer composition changes, while in northeastern Ohio, such increases drove composition shifts. U.S. temperatures are rising faster than the global average, projected to increase by 2.4°C–3.1°C (Marvel et al. 2023, NCA5). In the Midwest, annual temperatures have risen since the early 20th century and are expected to continue increasing (Wilson et al. 2023, NCA5). Accelerated warming has major consequences for wildlife movement, ecosystem functions, and agriculture, which can be further complicated by the changing land cover and use.

**FIGURE 10:**
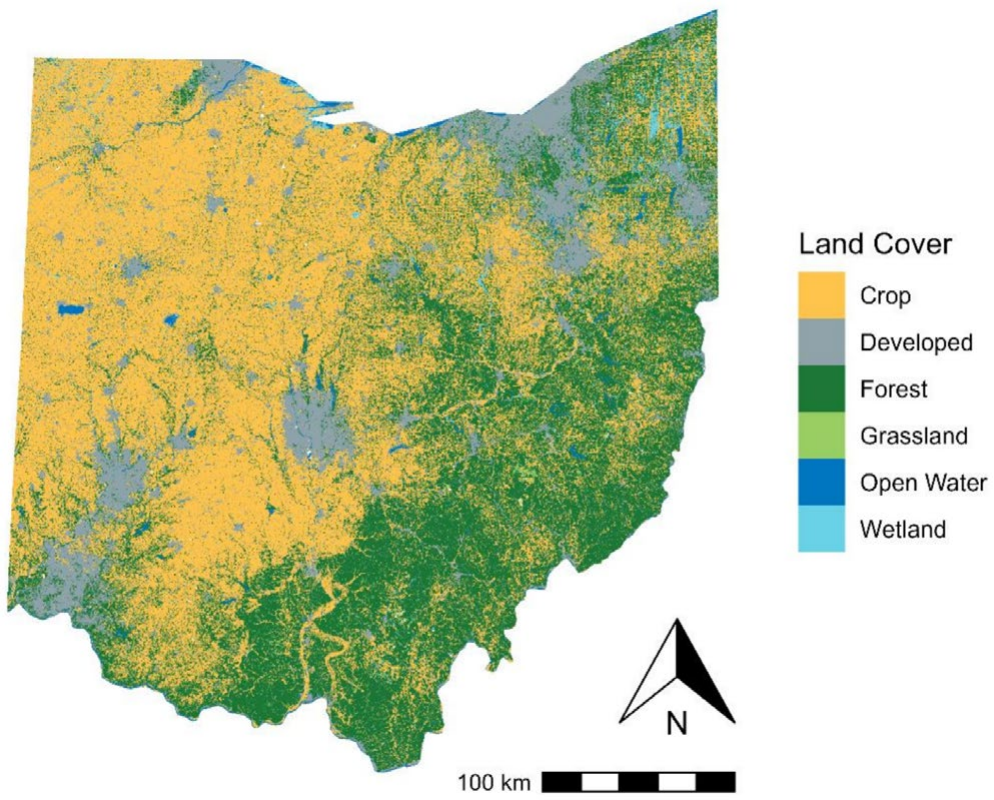
Ohio land cover in 2011 (end of the second Ohio Breeding Bird Atlas survey period). Developed class includes developed open space, low, medium, and high intensity. Forest class includes deciduous, evergreen, mixed, shrub, and herbaceous. Cultivated crops class includes hay, pasture, and cultivation. Wetlands class includes woody and emergent herbaceous wetlands.

Land uses have long‐lasting impacts on biotic interactions (biological legacies, Dupouey et al. 2024) and spatial structures. Western Ohio's landscape is dominated by agricultural land use (Figure 10), a transformation driven by post‐World War II technological advances and policy incentives like the New Deal (Bachman 1952; Krause and Kyle 1970; Lobao and Meyer 2001; Fry et al. 2011). This shift replaced mixed pastures and small farms with large‐scale monocultures (e.g., corn and soybeans), fragmenting forest habitats and homogenizing the land cover (Medley et al. 1995; Fry et al. 2011). Notably, our clusters of high temporal dissimilarity—blocks with substantial composition change—align with these agricultural‐intensive regions, where total forest loss has been severe (Figure S5) and elevation variability is minimal (Figure S6), highlighting the impact of human disturbance and large‐scale homogeneous crop covers, especially when there is no topographic buffering. These clusters of high dissimilarity values, identified with local indicators of spatial associations (LISA), suggest the spatial dependence of forest bird species composition shifts in this region, such that the reorganization of assemblages in one block propagates to neighboring blocks. This pattern implies that disturbances in one area may cascade across adjacent communities, amplifying instability. The spatial synchrony of high‐dissimilarity blocks further suggests that conservation efforts targeting isolated forest patches may be insufficient; instead, landscape‐scale habitat networks are needed to disrupt disturbance propagation and maintain regional biodiversity.

In contrast, forest bird communities in southeastern Ohio exhibit lower temporal dissimilarity, reflecting greater long‐term stability. This pattern reflects the role that ecological refugia play, shaped by its unique land‐use history and topographic complexity (Rossetto and Kooyman 2021). Large‐scale forest conversion in Ohio—which reduced statewide forest cover from ~95% pre‐colonization (1788) to 20% by 1910 (Dyer 2001; Smith 1977). In southeastern Ohio, following large‐scale forest clearing in association with iron production, the region's steep slopes and elevation variability discouraged intensive agriculture due to high transportation costs and low crop yields (Beatty and Stone 1984), leading to widespread farmland abandonment and forest regeneration by the mid‐20th century (Monsted and Matlack 2021). The mature, continuous forests (Figure S5) and pronounced elevation variation (Figure S6) in southeastern Ohio create microhabitats that buffer against regional environmental changes (Betts et al. 2018). Forest cover mitigates sun exposure, minimizing temperature fluctuations and controlling water evapotranspiration, which cools the atmosphere (Betts et al. 2018). Local terrain features, such as elevation, influence air temperature, precipitation, and solar radiation, decoupling from regional climatic trends (Thornthwaite 1954; Daly et al. 2010; Dobrowski 2011). Elevation variability introduces vertical and three‐dimensional space, fostering spatial and topographic complexity. This variability creates climatic gradients, leading to mesic or xeric moisture conditions (Dobrowski 2011; Byrne et al. 2017). Furthermore, the pronounced elevation variability fosters niche partitioning and limits human activity, enhancing habitat stability. The region's combination of large, connected forest patches and topographic complexity provides features absent elsewhere in the state. These conditions create diverse microhabitats that buffer forest avian communities against climate variability and human disturbance, promoting long‐term stability.

Ecological community studies often aim to understand the relationships between biotic dynamics (e.g., diversity) and environmental conditions. Ecological studies often assume the relationships between species dynamics and environmental factors are spatially stationary within the study area (Mcnew et al. 2013; Ye et al. 2020). However, in heterogeneous landscapes, the species‐environment relationships may be confounded by spatially varying trends that, if not carefully investigated, may lead to misinterpretation of the true factors that influence the observed patterns and biased ecological inference (Mcnew et al. 2013; Ye et al. 2020; Rollinson et al. 2021). Our GWR results reveal that the direction and magnitude of the forest community stability‐environment relationships are dynamic within Ohio, underscoring the need for spatially explicit approaches.

In eastern Ohio, dominated by development and residual forest (Flinn et al. 2018; McCluskey et al. 2018), forest bird communities become more unstable as annual maximum temperatures increase, and this trend is expected to continue with further temperature increases. Conversely, in northwestern Ohio, forest bird communities experience less compositional change with increasing annual maximum temperatures, aligning with the species pool hypothesis (Pärtel et al. 1996) and environmental filtering (Srivastava 1999; Kraft et al. 2015; Cadotte and Tucker 2017). In northwestern Ohio, homogeneous agricultural crops have dominated since the mid‐20th century, favoring forest species adapted to conditions such as low vegetation cover and elevated temperatures.

One interesting finding is the seemingly contradictory patterns between temporal dissimilarity and changes in total forest area versus temporal dissimilarity and changes in forest mean patch size in western Ohio. Temporal dissimilarity and changes in total forest area are negatively correlated; as total forest area increases, temporal dissimilarity decreases, suggesting that gaining total forest area facilitates forest avian community stability. On the contrary, in the same area, temporal dissimilarity and changes in forest mean patch size are positively correlated; when the average forest patch size increases, temporal dissimilarity also increases, indicating that the bigger the forest patches are, the more unstable the communities become. The key to untangling this contradiction is that the mean patch size metric only represents the average condition, but it does not convey information about the numbers of patches (McGarigal and Marks 1995). For example, a mean patch size of 10 ha could be either one 10 ha patch or a landscape with 5, 5, 10, 10, 15, 25 ha patches. Therefore, this metric alone offers limited information regarding landscape patterns. When combined with total forest area, the interpretation becomes clearer. Multiple lines of evidence suggest that habitat amount within a landscape has a positive relationship with biodiversity (Haddad et al. 2015; Watling et al. 2020), and our study lends one more support to this hypothesis. In western Ohio, increases in total forest area are associated with reduced compositional dissimilarity. If gaining total forest area facilitates community stability, why does the increase in average forest patch size link to instability? We suspect that this pattern points to habitat fragmentation. Forests in western Ohio are few, large, but isolated patches. Many studies have shown that habitat fragmentation has negative consequences on biodiversity (see Fletcher et al. 2018, 2023). Notably, this fragmentation signal was detectable only through GWR, as global models (e.g., Random Forest) ranked edge density low—a testament to spatial heterogeneity's role in masking local ecological processes.

Our use of elevation variability (coefficient of variation of elevation) as a predictor, rather than absolute elevation values, to prioritize topographic heterogeneity's role in shaping microhabitats (Dobrowski 2011; Suggitt et al. 2018; Navarro‐Serrano et al. 2020). The absolute elevation range in Ohio is less than 600 m (104–473 m), yet at local scales the variation in topography is a stronger determinant of microhabitat diversity than absolute elevation per se for temperate forest birds (Iverson et al. 2018; Adams et al. 2020). Furthermore, this approach allows direct comparison across Ohio's regions.

Our study demonstrates that large, connected forest patches and topographic complexity are critical for stabilizing temperate forest avian communities, buffering them against regional environmental change. By creating microhabitats and niche diversity, these landscape features reduce compositional shifts—a finding with urgent implications for conservation in fragmented habitats. Importantly, we reveal that spatial heterogeneity underpins species‐environment relationships with drivers like maximum temperature exerting opposing effects in different regions. As the first study to leverage Breeding Bird Atlas data for analyzing avian community dynamics, we provide a template for extracting novel insights from long‐term participatory science datasets. Our approach—prioritizing beta diversity and spatially explicit models before species‐level analysis—offers a powerful framework to identify conservation hotspots and diagnose ecosystem vulnerability. Future efforts could extend this work by integrating functional traits, but our results already underscore that preserving landscape heterogeneity is paramount to safeguarding avian biodiversity in the Anthropocene.

## Author Contributions

**Jess Dong:** conceptualization (lead), data curation (equal), formal analysis (lead), methodology (lead), visualization (lead), writing – original draft (lead). **Stephen N. Matthews:** conceptualization (supporting), supervision (lead), writing – review and editing (equal). **Matthew B. Shumar:** data curation (equal), validation (equal), writing – review and editing (equal). **William E. Peterman:** conceptualization (supporting), writing – review and editing (equal).

## Conflicts of Interest

The authors declare no conflicts of interest.

## Supporting information


**Appendix S1:** ece372359‐sup‐0001‐AppendixS1.docx.

## Data Availability

The data that support the findings of this study are openly available in Github at https://github.com/JessciaH‐D/forest‐community‐diversity and their references and sources are given in the manuscript.

## References

[ece372359-bib-0001] Adams, B. , L. Iverson , S. Matthews , M. Peters , A. Prasad , and D. M. Hix . 2020. “Mapping Forest Composition With Landsat Time Series: An Evaluation of Seasonal Composites and Harmonic Regression.” Remote Sensing 12: 610. 10.3390/rs12040610.

[ece372359-bib-0003] Akima, H. , and A. Gebhardt . 2022. “akima: Interpolation of Irregularly and Regularly Spaced Data.” https://CRAN.R‐project.org/package=akima.

[ece372359-bib-0004] Bachman, K. L. 1952. “Changes in Scale in Commercial Farming and Their Implications.” Journal of Farm Economics 34, no. 2: 157. 10.2307/1233339.

[ece372359-bib-0005] Badgley, C. , T. M. Smiley , R. Terry , et al. 2017. “Biodiversity and Topographic Complexity: Modern and Geohistorical Perspectives.” Trends in Ecology & Evolution 32, no. 3: 211–226. 10.1016/j.tree.2016.12.010. 28196688 PMC5895180

[ece372359-bib-0006] Baselga, A. , D. Orme , S. Villeger , et al. 2023. “betapart: Partitioning Beta Diversity into Turnover and Nestedness Components.” https://CRAN.R‐project.org/package=betapart.

[ece372359-bib-0008] Bateman, B. L. , L. Taylor , C. Wilsey , J. Wu , G. S. LeBaron , and G. Langham . 2020. “Risk to North American Birds From Climate Change‐Related Threats.” Conservation Science and Practice 2, no. 8: e243. 10.1111/csp2.243.

[ece372359-bib-0010] Beatty, E. G. , and M. S. Stone . 1984. Getting to Know Athens County. Stone House Publications.

[ece372359-bib-0011] Bellard, C. , C. Bertelsmeier , P. Leadley , W. Thuiller , and F. Courchamp . 2012. “Impacts of Climate Change on the Future of Biodiversity: Biodiversity and Climate Change.” Ecology Letters 15, no. 4: 365–377. 10.1111/j.1461-0248.2011.01736.x. 22257223 PMC3880584

[ece372359-bib-0012] Betts, M. G. , B. Phalan , S. J. K. Frey , J. S. Rousseau , and Z. Yang . 2018. “Old‐Growth Forests Buffer Climate‐Sensitive Bird Populations From Warming.” Diversity and Distributions 24, no. 4: 439–447. 10.1111/ddi.12688.

[ece372359-bib-0013] Bivand, R. S. , and D. W. S. Wong . 2018. “Comparing Implementations of Global and Local Indicators of Spatial Association.” Test 27, no. 3: 716–748. 10.1007/s11749-018-0599-x.

[ece372359-bib-0014] Borges, F. , M. Glemnitz , A. Schultz , and U. Stachow . 2017. “Assessing the Habitat Suitability of Agricultural Landscapes for Characteristic Breeding Bird Guilds Using Landscape Metrics.” Environmental Monitoring and Assessment 189, no. 4: 166. 10.1007/s10661-017-5837-2. 28303521 PMC5355513

[ece372359-bib-0015] Breiman, L. 2001. “Random Forests.” Machine Learning 45, no. 1: 5–32. 10.1023/A:1010933404324.

[ece372359-bib-0016] Bueno de Mesquita, C. P. , C. T. White , E. C. Farrer , L. M. Hallett , and K. N. Suding . 2021. “Taking Climate Change Into Account: Non‐Stationarity in Climate Drivers of Ecological Response.” Journal of Ecology 109, no. 3: 1491–1500. 10.1111/1365-2745.13572.

[ece372359-bib-0017] Byrne, M. , M. A. Millar , D. J. Coates , et al. 2017. “Refining Expectations for Environmental Characteristics of Refugia: Two Ranges of Differing Elevation and Topographical Complexity Are Mesic Refugia in an Arid Landscape.” Journal of Biogeography 44, no. 11: 2539–2550. 10.1111/jbi.13057.

[ece372359-bib-0018] Cadotte, M. W. , and C. M. Tucker . 2017. “Should Environmental Filtering Be Abandoned?” Trends in Ecology & Evolution 32, no. 6: 429–437. 10.1016/j.tree.2017.03.004. 28363350

[ece372359-bib-0019] Cardinale, B. J. , J. E. Duffy , A. Gonzalez , et al. 2012. “Biodiversity Loss and Its Impact on Humanity.” Nature 486, no. 7401: 59–67. 10.1038/nature11148. 22678280

[ece372359-bib-0021] Chao, A. , S. Thorn , C.‐. H. Chiu , et al. 2023. “Rarefaction and Extrapolation With Beta Diversity Under a Framework of Hill Numbers: The iNEXT.beta3D Standardization.” Ecological Monographs 93, no. 4: e1588. 10.1002/ecm.1588.

[ece372359-bib-0022] Clarke, A. , and K. J. Gaston . 2006. “Climate, Energy and Diversity.” Proceedings of the Biological Sciences 273, no. 1599: 2257–2266. 10.1098/rspb.2006.3545. 16928626 PMC1636092

[ece372359-bib-0023] Coops, N. C. , M. A. Wulder , and D. Iwanicka . 2009. “Exploring the Relative Importance of Satellite‐Derived Descriptors of Production, Topography and Land Cover for Predicting Breeding Bird Species Richness Over Ontario, Canada.” Remote Sensing of Environment 113, no. 3: 668–679. 10.1016/j.rse.2008.11.012.

[ece372359-bib-0024] Cornell, H. V. , and S. P. Harrison . 2014. “What Are Species Pools and When Are They Important?” Annual Review of Ecology, Evolution, and Systematics 45, no. 1: 45–67. 10.1146/annurev-ecolsys-120213-091759.

[ece372359-bib-0025] Dahirel, M. , A. Bertin , M. Haond , et al. 2021. “Shifts From Pulled to Pushed Range Expansions Caused by Reduction of Landscape Connectivity.” Oikos 130, no. 5: 708–724. 10.1111/oik.08278.

[ece372359-bib-0026] Daly, C. , D. R. Conklin , and M. H. Unsworth . 2010. “Local Atmospheric Decoupling in Complex Topography Alters Climate Change Impacts.” International Journal of Climatology 30, no. 12: 1857–1864. 10.1002/joc.2007.

[ece372359-bib-0028] Díaz, S. , and Y. Malhi . 2022. “Biodiversity: Concepts, Patterns, Trends, and Perspectives.” Annual Review of Environment and Resources 47, no. 1: 31–63. 10.1146/annurev-environ-120120-054300.

[ece372359-bib-0029] Dobrowski, S. Z. 2011. “A Climatic Basis for Microrefugia: The Influence of Terrain on Climate.” Global Change Biology 17, no. 2: 1022–1035. 10.1111/j.1365-2486.2010.02263.x.

[ece372359-bib-0030] Donaldson, M. R. , N. J. Burnett , D. C. Braun , et al. 2017. “Taxonomic Bias and International Biodiversity Conservation Research.” Facets 1: 105–113. 10.1139/facets-2016-0011.

[ece372359-bib-0031] Dornelas, M. , N. J. Gotelli , B. McGill , et al. 2014. “Assemblage Time Series Reveal Biodiversity Change but Not Systematic Loss.” Science 344, no. 6181: 296–299. 10.1126/science.1248484. 24744374

[ece372359-bib-0032] Dray, S. , D. Bauman , G. Blanchet , et al. 2023. “adespatial: Multivariate Multiscale Spatial Analysis.” https://CRAN.R‐project.org/package=adespatial.

[ece372359-bib-0033] Dray, S. , P. Legendre , and P. R. Peres‐Neto . 2006. “Spatial Modelling: A Comprehensive Framework for Principal Coordinate Analysis of Neighbour Matrices (PCNM).” Ecological Modelling 196, no. 3–4: 483–493. 10.1016/j.ecolmodel.2006.02.015.

[ece372359-bib-0034] Dupouey, J. L. , E. Dambrine , J. D. Laffite , and C. Moares . 2024. “Irreversible Impact of Past Land Use on Forest Soils and Biodiversity.” 83, no. 11: 2978–2984.

[ece372359-bib-0035] Dyer, J. M. 2001. “Using Witness Trees to Assess Forest Change in Southeastern Ohio.” Canadian Journal of Forest Research 31, no. 10: 1708–1718. 10.1139/x01-111.

[ece372359-bib-0036] Evans, J. S. , M. A. Murphy , Z. A. Holden , and S. A. Cushman . 2011. “Modeling Species Distribution and Change Using Random Forest.” In Predictive Species and Habitat Modeling in Landscape Ecology: Concepts and Applications, edited by C. A. Drew , Y. F. Wiersma , and F. Huettmann , 139–159. Springer. 10.1007/978-1-4419-7390-0_8.

[ece372359-bib-0114] Fischer, A. , P. Marshall , and A. Camp . 2013. “Disturbances in Deciduous Temperate Forest Ecosystems of the Northern Hemisphere: Their Effects on Both Recent and Future Forest Development.” Biodiversity and Conservation 22, no. 9: 1863–1893. 10.1007/s10531-013-0525-1.

[ece372359-bib-0037] Fletcher, R. J. , M. G. Betts , E. I. Damschen , et al. 2023. “Addressing the Problem of Scale That Emerges With Habitat Fragmentation.” Global Ecology and Biogeography 32, no. 6: 828–841. 10.1111/geb.13658.

[ece372359-bib-0038] Fletcher, R. J. , R. K. Didham , C. Banks‐Leite , et al. 2018. “Is Habitat Fragmentation Good for Biodiversity?” Biological Conservation 226: 9–15. 10.1016/j.biocon.2018.07.022.

[ece372359-bib-0039] Flinn, K. M. , T. P. Mahany , and C. E. Hausman . 2018. “From Forest to City: Plant Community Change in Northeast Ohio From 1800 to 2014.” Journal of Vegetation Science 29, no. 2: 297–306. 10.1111/jvs.12621.

[ece372359-bib-0040] Frey, S. J. K. , A. S. Hadley , S. L. Johnson , M. Schulze , J. A. Jones , and M. G. Betts . 2016. “Spatial Models Reveal the Microclimatic Buffering Capacity of Old‐Growth Forests.” Science Advances 2, no. 4: e1501392. 10.1126/sciadv.1501392. 27152339 PMC4846426

[ece372359-bib-0041] Fry, J. , G. Z. Xian , S. Jin , et al. 2011. “Completion of the 2006 National Land Cover Database for the Conterminous United States.” Photogrammetric Engineering & Remote Sensing 77, no. 9: 858–864.

[ece372359-bib-0112] Gilliam, F. S. 2016. “Forest Ecosystems of Temperate Climatic Regions: From Ancient Use to Climate Change.” New Phytologist 212, no. 4: 871–887. 10.1111/nph.14255. 27787948

[ece372359-bib-0042] Gollini, I. , B. Lu , M. Charlton , C. Brunsdon , and P. Harris . 2015. “GWmodel: An R Package for Exploring Spatial Heterogeneity Using Geographically Weighted Models.” Journal of Statistical Software 63: 1–50. 10.18637/jss.v063.i17.

[ece372359-bib-0043] Goward, S. N. , C. Huang , F. Zhao , et al. 2016. NACP NAFD Project: Forest Disturbance History From Landsat, 1986‐2010. ORNL DAAC. 10.3334/ORNLDAAC/1290.

[ece372359-bib-0044] Greenwell, B. M. 2017. “Pdp: An R Package for Constructing Partial Dependence Plots.” R Journal 9, no. 1: 421. 10.32614/RJ-2017-016.

[ece372359-bib-0045] Greiser, C. , J. Ehrlén , E. Meineri , and K. Hylander . 2020. “Hiding From the Climate: Characterizing Microrefugia for Boreal Forest Understory Species.” Global Change Biology 26, no. 2: 471–483. 10.1111/gcb.14874. 31833152 PMC7027894

[ece372359-bib-0046] Haddad, N. M. , L. A. Brudvig , J. Clobert , et al. 2015. “Habitat Fragmentation and Its Lasting Impact on Earth's Ecosystems.” Science Advances 1, no. 2: e1500052. 10.1126/sciadv.1500052. 26601154 PMC4643828

[ece372359-bib-0047] Hampe, A. , and A. S. Jump . 2011. “Climate Relicts: Past, Present, Future.” Annual Review of Ecology, Evolution, and Systematics 42: 313–333.

[ece372359-bib-0048] Hesselbarth, M. H. K. , M. Sciaini , K. A. With , K. Wiegand , and J. Nowosad . 2019. “Landscapemetrics: An Open‐Source R Tool to Calculate Landscape Metrics.” Ecography 42, no. 10: 1648–1657. 10.1111/ecog.04617.

[ece372359-bib-0049] Hijmans, R. J. 2023. “terra: Spatial Data Analysis.” https://CRAN.R‐project.org/package=terra.

[ece372359-bib-0050] Hillebrand, H. , B. Blasius , E. T. Borer , et al. 2018. “Biodiversity Change Is Uncoupled From Species Richness Trends: Consequences for Conservation and Monitoring.” Journal of Applied Ecology 55, no. 1: 169–184. 10.1111/1365-2664.12959.

[ece372359-bib-0051] Hornung, R. , and M. N. Wright . 2023. “diversityForest: Innovative Complex Split Procedures in Random Forests Through Candidate Split Sampling.” https://CRAN.R‐project.org/package=diversityForest. 10.1007/s42979-021-00920-1 PMC8533673 34723205

[ece372359-bib-0052] Iverson, L. R. , M. P. Peters , J. L. Bartig , et al. 2018. “Spatial Modeling and Inventories for Prioritizing Investment Into Oak‐Hickory Restoration.” Forest Ecology and Management 424: 355–366. 10.1016/j.foreco.2018.05.018.

[ece372359-bib-0053] Jarzyna, M. A. , W. F. Porter , B. A. Maurer , B. Zuckerberg , and A. O. Finley . 2015. “Landscape Fragmentation Affects Responses of Avian Communities to Climate Change.” Global Change Biology 21, no. 8: 2942–2953. 10.1111/gcb.12885. 25644514

[ece372359-bib-0054] Jiguet, F. , R. Julliard , D. Couvet , and A. Petiau . 2005. “Modeling Spatial Trends in Estimated Species Richness Using Breeding Bird Survey Data: A Valuable Tool in Biodiversity Assessment.” Biodiversity and Conservation 14, no. 13: 3305–3324. 10.1007/s10531-004-0448-y.

[ece372359-bib-0056] Kraft, N. J. B. , P. B. Adler , O. Godoy , E. C. James , S. Fuller , and J. M. Levine . 2015. “Community Assembly, Coexistence and the Environmental Filtering Metaphor.” Functional Ecology 29, no. 5: 592–599. 10.1111/1365-2435.12345.

[ece372359-bib-0057] Krause, K. R. , and L. R. Kyle . 1970. “Economic Factors Underlying the Incidence of Large Farming Units: The Current Situation and Probable Trends.” American Journal of Agricultural Economics 52, no. 5: 748–761. 10.2307/1237697.

[ece372359-bib-0058] Lamy, T. , P. Legendre , Y. Chancerelle , G. Siu , and J. Claudet . 2015. “Understanding the Spatio‐Temporal Response of Coral Reef Fish Communities to Natural Disturbances: Insights From Beta‐Diversity Decomposition.” PLoS One 10, no. 9: e0138696. 10.1371/journal.pone.0138696. 26393511 PMC4578945

[ece372359-bib-0059] Leech, D. I. , and H. Q. P. Crick . 2007. “Influence of Climate Change on the Abundance, Distribution and Phenology of Woodland Bird Species in Temperate Regions.” Ibis 149, no. s2: 128–145. 10.1111/j.1474-919X.2007.00729.x.

[ece372359-bib-0060] Legendre, P. , and M. J. Fortin . 1989. “Spatial Pattern and Ecological Analysis.” Vegetatio 80, no. 2: 107–138. 10.1007/BF00048036.

[ece372359-bib-0061] Lenoir, J. , T. Hattab , and G. Pierre . 2017. “Climatic Microrefugia Under Anthropogenic Climate Change: Implications for Species Redistribution.” Ecography 40, no. 2: 253–266. 10.1111/ecog.02788.

[ece372359-bib-0062] Leung, Y. , C. Mei , and W.‐X. Zhang . 2000. “Statistical Tests for Spatial Nonstationary Based on the Geographically Weighted Regression Model.” Environment & Planning A 32: 9–32. 10.1068/a3162.

[ece372359-bib-0063] Levin, S. A. 1992. “The Problem of Pattern and Scale in Ecology: The Robert H. MacArthur Award Lecture.” Ecology 73, no. 6: 1943–1967. 10.2307/1941447.

[ece372359-bib-0064] Liaw, A. , and M. Wiener . 2002. “Classification and Regression by randomForest.” R News 2: 18–22.

[ece372359-bib-0065] Lobao, L. , and K. Meyer . 2001. “The Great Agricultural Transition: Crisis, Change, and Social Consequences of Twentieth Century US Farming.” Annual Review of Sociology 27, no. 1: 103–124. 10.1146/annurev.soc.27.1.103.

[ece372359-bib-0066] Loreau, M. , and C. De Mazancourt . 2013. “Biodiversity and Ecosystem Stability: A Synthesis of Underlying Mechanisms.” Ecology Letters 16, no. s1: 106–115. 10.1111/ele.12073. 23346947

[ece372359-bib-0067] Lu, B. , P. Harris , M. Charlton , and C. Brunsdon . 2014. “The GWmodel R Package: Further Topics for Exploring Spatial Heterogeneity Using Geographically Weighted Models.” Geo‐Spatial Information Science 17, no. 2: 85–101. 10.1080/10095020.2014.917453.

[ece372359-bib-0068] Magurran, A. E. , M. Dornelas , F. Moyes , and P. A. Henderson . 2019. “Temporal β Diversity—A Macroecological Perspective Storch D, Editor.” Global Ecology and Biogeography 28, no. 12: 1949–1960. 10.1111/geb.13026.

[ece372359-bib-0069] Marvel, K. , W. Su , R. Delgado , et al. 2023. “Climate Trends.” In Fifth National Climate Assessment. U.S. Global Change Research Program. https://nca2023.globalchange.gov/chapter/2/.

[ece372359-bib-0070] McCluskey, E. M. , S. N. Matthews , I. Y. Ligocki , M. L. Holding , G. J. Lipps , and T. E. Hetherington . 2018. “The Importance of Historical Land Use in the Maintenance of Early Successional Habitat for a Threatened Rattlesnake.” Global Ecology and Conservation 13: e00370. 10.1016/j.gecco.2017.e00370.

[ece372359-bib-0071] McGarigal, K. , and B. J. Marks . 1995. FRAGSTATS: Spatial Pattern Analysis Program for Quantifying Landscape Structure. Gen Tech Rep PNW‐GTR‐351, 351. US Dep Agric For Serv Pac Northwest Res Stn 122. https://research.fs.usda.gov/treesearch/3064.

[ece372359-bib-0072] Mcnew, L. B. , A. J. Gregory , and B. K. Sandercock . 2013. “Spatial Heterogeneity in Habitat Selection: Nest Site Selection by Greater Prairie‐Chickens.” Journal of Wildlife Management 77, no. 4: 791–801. 10.1002/jwmg.493.

[ece372359-bib-0073] Medley, K. E. , B. W. Okey , G. W. Barrett , M. F. Lucas , and W. H. Renwick . 1995. “Landscape Change With Agricultural Intensification in a Rural Watershed, Southwestern Ohio, USA.” Landscape Ecology 10, no. 3: 161–176. 10.1007/BF00133029.

[ece372359-bib-0074] Monsted, J. , and G. R. Matlack . 2021. “Shaping the Second‐Growth Forest: Fine‐Scale Land Use Change in the Ohio Valley Over 120 Years.” Landscape Ecology 36, no. 12: 3507–3521. 10.1007/s10980-021-01323-6.

[ece372359-bib-0075] Morelli, F. , Y. Benedetti , and P. Šímová . 2018. “Landscape Metrics as Indicators of Avian Diversity and Community Measures.” Ecological Indicators 90: 132–141. 10.1016/j.ecolind.2018.03.011.

[ece372359-bib-0076] Navarro‐Serrano, F. , J. I. López‐Moreno , C. Azorin‐Molina , et al. 2020. “Elevation Effects on Air Temperature in a Topographically Complex Mountain Valley in the Spanish Pyrenees.” Atmosphere 11, no. 6: 656. 10.3390/atmos11060656.

[ece372359-bib-0077] Newbold, T. , G. L. Adams , G. Albaladejo Robles , et al. 2019. “Climate and Land‐Use Change Homogenise Terrestrial Biodiversity, With Consequences for Ecosystem Functioning and Human Well‐Being.” Emerging Topics in Life Sciences 3, no. 2: 207–219. 10.1042/ETLS20180135. 33523149

[ece372359-bib-0078] Newbold, T. , L. N. Hudson , S. L. L. Hill , et al. 2015. “Global Effects of Land Use on Local Terrestrial Biodiversity.” Nature 520, no. 7545: 45–50. 10.1038/nature14324. 25832402

[ece372359-bib-0079] Northrup, J. M. , J. W. Rivers , Z. Yang , and M. G. Betts . 2019. “Synergistic Effects of Climate and Land‐Use Change Influence Broad‐Scale Avian Population Declines.” Global Change Biology 25, no. 5: 1561–1575. 10.1111/gcb.14571. 30810257

[ece372359-bib-0080] Oksanen, J. , G. L. Simpson , F. G. Blanchet , R. Kindt , and P. Legendre . 2022. “vegan:Community Ecology Package.” https://CRAN.R‐project.org/package=vegan.

[ece372359-bib-0081] Oliver, T. , D. B. Roy , J. K. Hill , T. Brereton , and C. D. Thomas . 2010. “Heterogeneous Landscapes Promote Population Stability.” Ecology Letters 13, no. 4: 473–484. 10.1111/j.1461-0248.2010.01441.x. 20148927

[ece372359-bib-0082] Pärtel, M. , M. Zobel , K. Zobel , E. Van Der Maarel , and M. Partel . 1996. “The Species Pool and Its Relation to Species Richness: Evidence From Estonian Plant Communities.” Oikos 75, no. 1: 111. 10.2307/3546327.

[ece372359-bib-0083] Pebesma, E. 2018. “Simple Features for R: Standardized Support for Spatial Vector Data.” R Journal 10, no. 1: 439–446.

[ece372359-bib-0084] Regos, A. , L. Imbeau , M. Desrochers , et al. 2018. “Hindcasting the Impacts of Land‐Use Changes on Bird Communities With Species Distribution Models of Bird Atlas Data.” Ecological Applications 28, no. 7: 1867–1883. 10.1002/eap.1784. 30055061

[ece372359-bib-0115] Riitters, K. H. , J. W. Coulston , and J. D. Wickham . 2012. “Fragmentation of Forest Communities in the Eastern United States.” Forest Ecology and Management 263: 85–93. 10.1016/j.foreco.2011.09.022.

[ece372359-bib-0085] Rodewald, P. G. , M. B. Shumar , A. T. Boone , D. L. Slager , and J. McCormac . 2016. The Second Atlas of Breeding Birds in Ohio. Pennsylvania State University Press.

[ece372359-bib-0086] Rollinson, C. R. , A. O. Finley , M. R. Alexander , et al. 2021. “Working Across Space and Time: Nonstationarity in Ecological Research and Application.” Frontiers in Ecology and the Environment 19, no. 1: 66–72. 10.1002/fee.2298.

[ece372359-bib-0087] Rossetto, M. , and R. Kooyman . 2021. “Conserving Refugia: What Are we Protecting and Why?” Diversity 13, no. 2: 67. 10.3390/d13020067.

[ece372359-bib-0113] Rustad, L. , J. Campbell , J. S. Dukes , et al. 2012. “Changing Climate, Changing Forests: The Impacts of Climate Change on Forests of the Northeastern United States and Eastern Canada.” NRS‐GTR‐99. U.S. Department of Agriculture, Forest Service, Northern Research Station. 10.2737/NRS-GTR-99.

[ece372359-bib-0116] Sala, O. E. , F. I. Stuart Chapin , J. J. Armesto , et al. 2000. “Global Biodiversity Scenarios for the Year 2100.” Science 287, no. 5459: 1770–1774. 10.1126/science.287.5459.1770. 10710299

[ece372359-bib-0088] Saveraid, E. H. , D. M. Debinski , K. Kindscher , and M. E. Jakubauskas . 2001. “A Comparison of Satellite Data and Landscape Variables in Predicting Bird Species Occurrences in the Greater Yellowstone Ecosystem, USA.” Landscape Ecology 16, no. 1: 71–83. 10.1023/A:1008119219788.

[ece372359-bib-0090] Schloerke, B. , D. Cook , J. Larmarange , et al. 2024. “GGally: Extension to ‘ggplot2’.” https://CRAN.R‐project.org/package=GGally.

[ece372359-bib-0091] Sievert, C. 2020. Interactive Web‐Based Data Visualization With R, Plotly, and Shiny. Chapman and Hall/CRC. https://plotly‐r.com/.

[ece372359-bib-0092] Smith, T. H. 1977. The Mapping of Ohio. Kent State University Press.

[ece372359-bib-0093] Socolar, J. B. , J. J. Gilroy , W. E. Kunin , and D. P. Edwards . 2016. “How Should Beta‐Diversity Inform Biodiversity Conservation?” Trends in Ecology & Evolution 31, no. 1: 67–80. 10.1016/j.tree.2015.11.005. 26701706

[ece372359-bib-0094] Srivastava, D. S. 1999. “Using Local–Regional Richness Plots to Test for Species Saturation: Pitfalls and Potentials.” Journal of Animal Ecology 68, no. 1: 1–16. 10.1046/j.1365-2656.1999.00266.x.

[ece372359-bib-0095] Suggitt, A. J. , R. J. Wilson , N. J. B. Isaac , et al. 2018. “Extinction Risk From Climate Change Is Reduced by Microclimatic Buffering.” Nature Climate Change 8, no. 8: 713–717. 10.1038/s41558-018-0231-9.

[ece372359-bib-0097] Thornthwaite, C. W. 1954. “Topoclimatology.” In Proceedings of the Toronto Meterorological Conference, 227–232. Royal Meteorological Society.

[ece372359-bib-0098] Tobias, J. A. , C. Sheard , A. L. Pigot , et al. 2022. “AVONET: Morphological, Ecological and Geographical Data for All Birds.” Ecology Letters 25, no. 3: 581–597. 10.1111/ele.13898. 35199922

[ece372359-bib-0099] Tobler, W. R. 1970. “A Computer Movie Simulating Urban Growth in the Detroit Region.” Economic Geography 46: 234. 10.2307/143141.

[ece372359-bib-0100] Tolmos, M. L. , N. R. Guerrero‐Ramirez , A. Ameztegui , M. P. Barajas Barbosa , D. Craven , and H. Kreft . 2024. “Biogeographic Context Mediates Multifaceted Diversity‐Productivity Relationships in Island and Mainland Forests.” Journal of Ecology 112, no. 4: 800–816. 10.1111/1365-2745.14270.

[ece372359-bib-0101] Tsianou, M. A. , K. Touloumis , and A. S. Kallimanis . 2021. “Low Spatial Congruence Between Temporal Functional β‐Diversity and Temporal Taxonomic and Phylogenetic β‐Diversity in British Avifauna.” Ecological Research 36, no. 3: 491–505. 10.1111/1440-1703.12209.

[ece372359-bib-0102] Turner, M. G. 2005. “Landscape Ecology: What Is the State of the Science?” Annual Review of Ecology, Evolution, and Systematics 36: 319–344. 10.1146/annurev.ecolsys.36.102003.152614.

[ece372359-bib-0104] Watling, J. I. , V. Arroyo‐Rodríguez , M. Pfeifer , et al. 2020. “Support for the Habitat Amount Hypothesis From a Global Synthesis of Species Density Studies.” Ecology Letters 23, no. 4: 674–681. 10.1111/ele.13471. 32043741

[ece372359-bib-0105] Whittaker, R. H. 1960. “Vegetation of the Siskiyou Mountains, Oregon and California.” Ecological Monographs 30, no. 30: 279–338.

[ece372359-bib-0106] Whittaker, R. H. 1972. “Evolution and Measurement of Species Diversity.” Taxon 21, no. 2–3: 213–251. 10.2307/1218190.

[ece372359-bib-0107] Wiens, J. A. 2002. “Riverine Landscapes: Taking Landscape Ecology Into the Water.” Freshwater Biology 47, no. 4: 501–515. 10.1046/j.1365-2427.2002.00887.x.

[ece372359-bib-0108] Wilson, A. B. , J. M. Baker , E. A. Ainsworth , et al. 2023. “Midwest.” In Fifth National Climate Assessment. U.S. Global Change Research Program. https://nca2023.globalchange.gov/chapter/24/.

[ece372359-bib-0109] Wu, N. , Y. Wang , Y. Wang , X. Sun , C. Faber , and N. Fohrer . 2022. “Environment Regimes Play an Important Role in Structuring Trait‐ and Taxonomy‐Based Temporal Beta Diversity of Riverine Diatoms.” Journal of Ecology 110, no. 6: 1442–1454. 10.1111/1365-2745.13859.

[ece372359-bib-0110] Ye, X. , X. Yu , and T. Wang . 2020. “Investigating Spatial Non‐Stationary Environmental Effects on the Distribution of Giant Pandas in the Qinling Mountains, China.” Global Ecology and Conservation 21: e00894. 10.1016/j.gecco.2019.e00894.

