# Supplementary material for: Spatially Varying Drivers of Temporal β Diversity in Forest Avian Communities
Source: Ecol Evol. 2025 Oct 22;15(10):e72359. doi: 10.1002/ece3.72359 (PMC12542304; doi:10.1002/ece3.72359)
Supplement: Supplementary file 1 — Appendix S1: ece372359‐sup‐0001‐AppendixS1.docx. [file ECE3-15-e72359-s001.docx]

**Appendix**


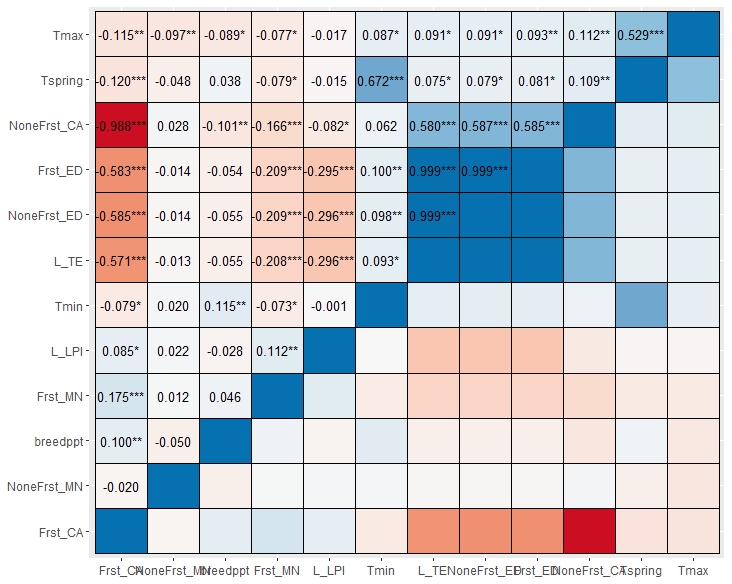


*Figure.1 Collinearity of climate and landscape variables for studying avian composition change in Ohio between 1982 and 2011. Climate variables include annual maximum temperature (Tmax), spring temperature (Tspring), annual minimum temperature (Tmin), and breeding season precipitation (breedppt). Landscape variables include forest edge density (frst_ED), forest mean patch size (frst_MN), forest core area (frst_CA), non-forest core area (NoneFrst_CA), non-forest edge density (NoneFrst_ED), non-forest mean patch size (NoneFrst_MN), total edge (TE), and largest patch index (LPI).*


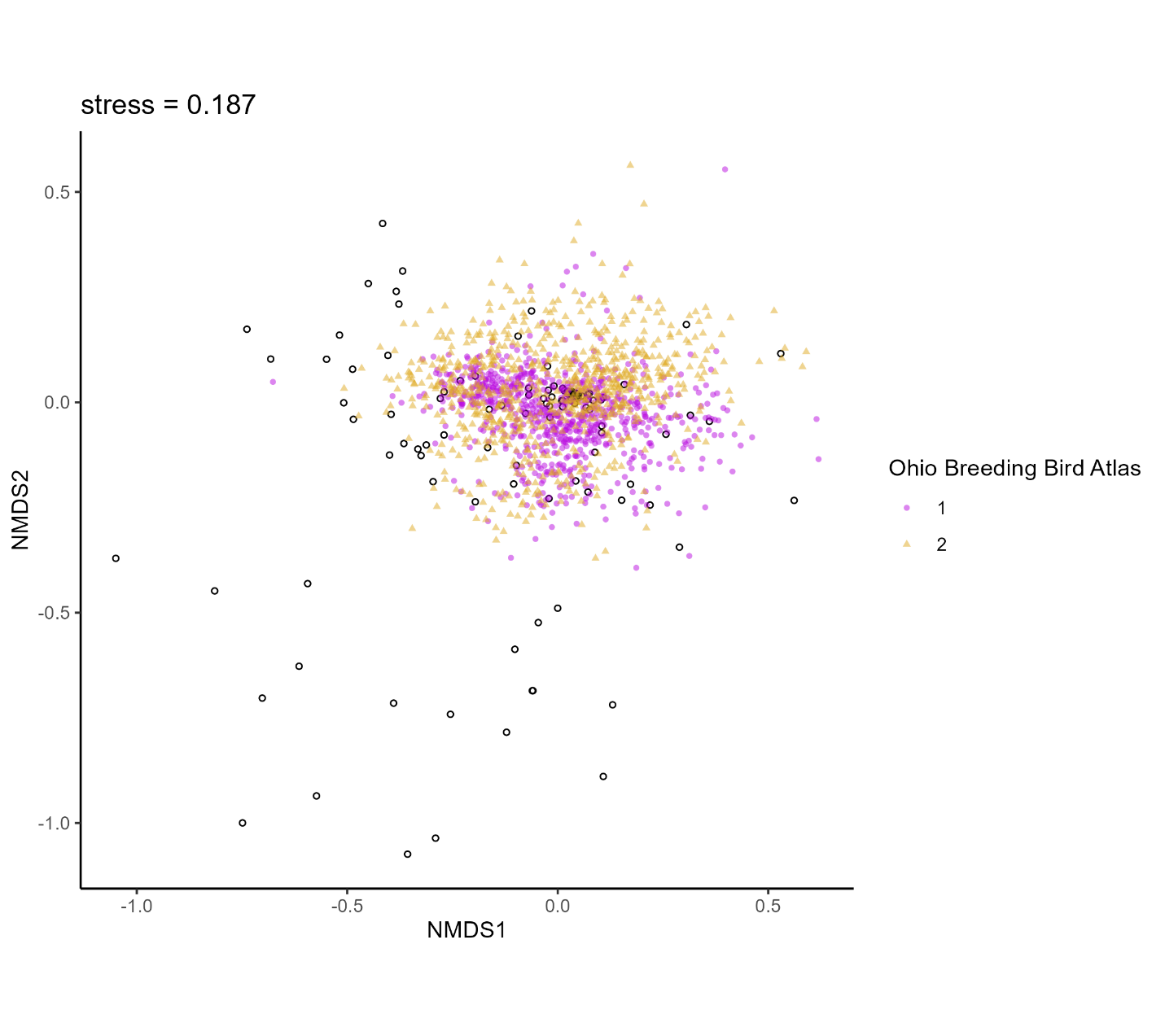


*Figure.2 Non-metric multidimensional scaling of Ohio avian community distances between 1982 and 2011. Purple represents the first sampling period (1982-1987) and yellow represents the second sampling period (2006-2011). Black circles represent the 113 species.*


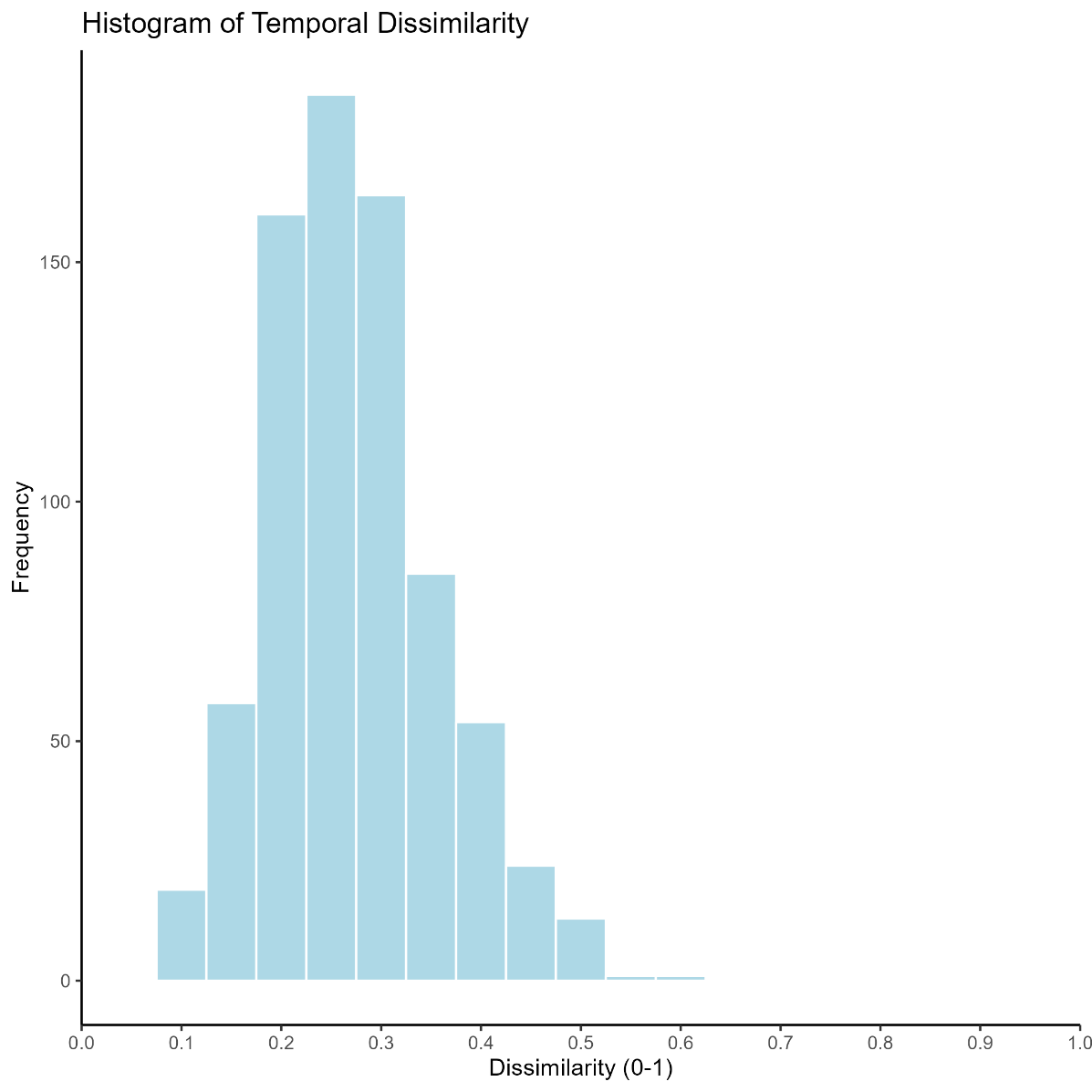


*Figure.3 Range values of the β diversity temporal dissimilarity (i.e. temporal dissimilarity) between Ohio Breeding Bird Atlas survey periods (1982-1987 & 2006-2011).*


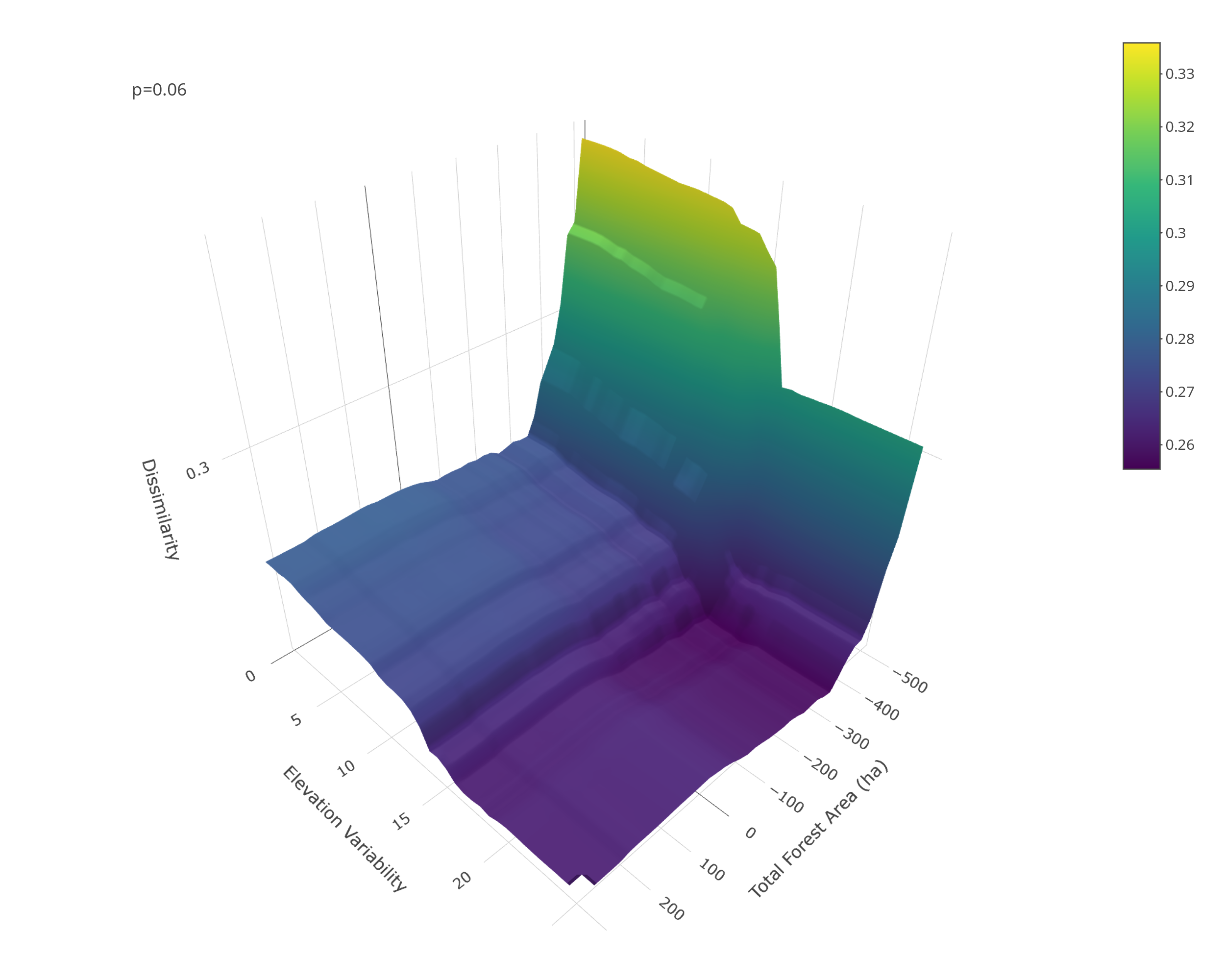


*Figure.4 Three-dimensional plot showing the weak interacting effects between elevation variability and total forest area on the temporal dissimilarity of β diversity, for the forest avian community in Ohio. Temporal dissimilarity, ranging from zero to one, was computed with the Jaccard index, based on the occurrence data from Ohio Breeding Bird Atlas in 1982-1987 and 2006-2011. Elevation variability was calculated as the coefficient of variation of elevation in each survey block. Total forest area was the sum of all forest patches in each survey block.*


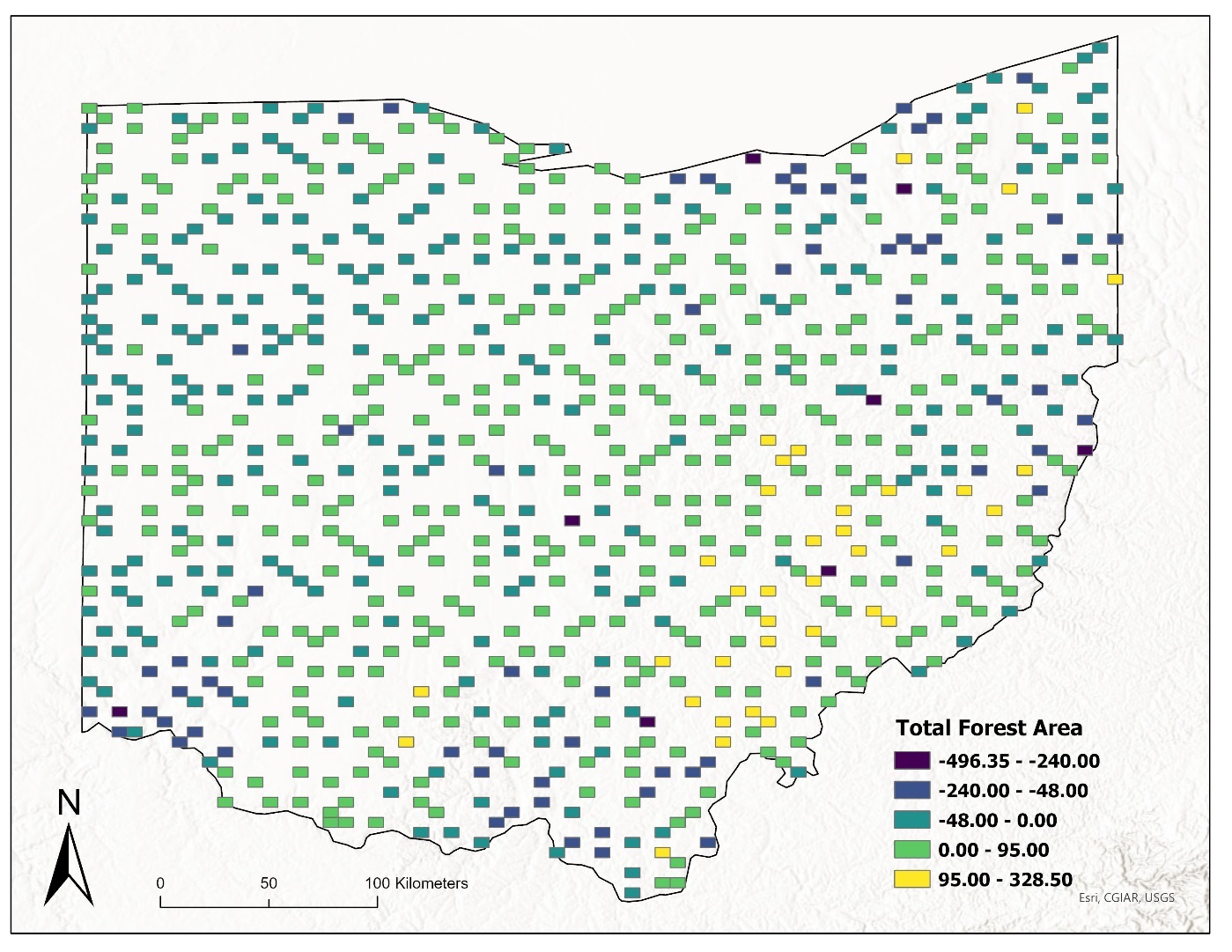


*Figure.5 Changes in total forest area between 1982 and 2011 in Ohio.*


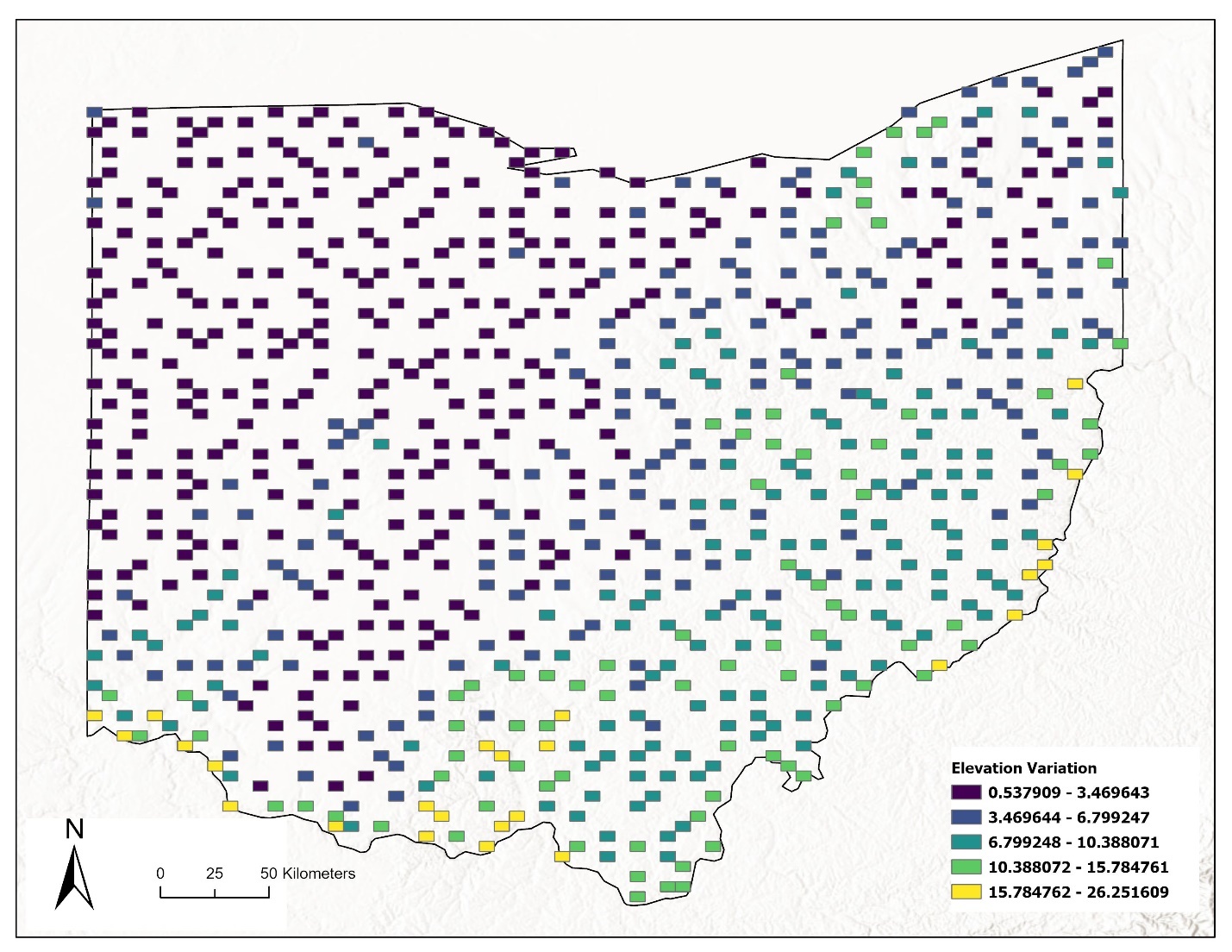


*Figure.6 Elevation variation in Ohio. Elevation variation was calculated as the coefficient variation (CV) of elevation without unit.*

Table.1 Landscape metrics that were used to derive landscape and class-level measurements of the Ohio Breeding Bird Atlas survey blocks.

| Level | Metric Name | Equation | Description | Class Level Metrics |
| --- | --- | --- | --- | --- |
| Class | Mean Patch Size (AREA_MN) | $MN=\frac{\sum_{i=1}^{m} \sum_{j=1}^{n} X_{ij}}{N}$  $AREA=a_{ij}\left( \frac{1}{10,000} \right)$ | MN equals the sum of corresponding patch values divided by the total number of patches.  AREA equals the area (m^2^) of patch ij divided by 10,000 to convert to hectares.  AREA_MN is calculated as the average area of patches of a given class within the landscape. | Forest mean patch size  Non-forest mean patch size  Water mean patch size* |
|  | Total Class Area  (CA) | $CA=\sum_{j=1}^{n} a_{ij}\left( \frac{1}{10,000} \right)$ | CA equals the sum of the areas of all the patches in the corresponding patch type, converted to hectares. | Total forest area  Total non-forest area*  Total water area* |
|  | Edge Density  (ED) | $ED= \frac{\sum_{k=1}^{m} e_{ik}}{A}\left( 10,000 \right)$ | ED equals the total length of edges of a corresponding patch type, divided by the total area (in hectares). Unit of ED is meters per hectare. | Forest edge density  Non-forest edge density*  Water edge density* |
| Landscape | Largest Patch Index  (LPI) | $LPI= \frac{max\left( a_{ij} \right)}{A}\left( 100 \right)$ | LPI equals the area of the largest patch in the landscape divided by the total landscape area, converted to a percentage. Unit of LPI is percent. | Largest Patch Index |
|  | Total Edge | *TE = E* | TE equals the total length of edge in a landscape. Unit of TE is meters. | Total Edge* |

*Metrics were excluded from the analyses due to high multicollinearity.

Table.2 The final set of environmental variables and range values used for understanding whether and how environmental changes affect temporal β diversity dissimilarity (i.e. temporal dissimilarity) in Ohio between 1986 and 2010. These variables were expressed as the change (∆) in values between the two time-points, except for Elevation Variability and Spatial Autocorrelation.

| Variable | Range of Value |
| --- | --- |
| Annual Maximum Temperature | -0.93 – 0.87 °C |
| Annual Minimum Temperature | -0.68 – 1.39 °C |
| Breeding Season Precipitation | -93.2 – 156.5 mm |
| Non-Forest Mean Patch Size | -1803.9 – 1551.4 ha |
| Forest Mean Patch Size | -439.1 – 1621.1 ha |
| Total Forest Area | -496.4 – 328.5 ha |
| Forest Edge Density | -25.6 – 26.0 m/ha |
| Largest Patch Index | -33.1 – 28.5 % |
| Elevation Variability | 0.54 – 26.25 |
| Spatial Autocorrelation | -0.0010 – 0.0013 |
